# Supplementary material for: Identification of a small-molecule inhibitor that selectively blocks DNA-binding by Trypanosoma brucei replication protein A1
Source: Nat Commun. 2023 Jul 20;14:4390. doi: 10.1038/s41467-023-39839-x (PMC10359466; doi:10.1038/s41467-023-39839-x)
Supplement: Supplementary file 1 — Supplementary Information [file 41467_2023_39839_MOESM1_ESM.pdf]

Supplementary Information

Identification of a small-molecule inhibitor that selectively blocks DNA-binding by *Trypanosoma brucei* Replication Protein A1

Aditi Mukherjee[1], Zakir Hossain[2], Esteban Erben[3,4], Shuai Ma[5], Jun Yong Choi[2,5,6,*] & Hee-Sook Kim[1,7,*]

[1]Public Health Research Institute, Rutgers Biomedical Health Sciences, Newark, NJ 07103, USA

[2]Department of Chemistry and Biochemistry, Queens College, New York, NY 11367, USA

[3]Instituto de Investigaciones Biotecnológicas, Universidad Nacional de San Martín (UNSAM) – Consejo Nacional de Investigaciones Científicas y Técnicas (CONICET), San Martín, Provincia de Buenos Aires, Argentina

[4]Escuela de Bio y Nanotecnologías (EByN), Universidad Nacional de San Martín, San Martín, Provincia de Buenos Aires, Argentina

[5]Ph.D. Programs in Chemistry, The Graduate Center of the City University of New York, New York, NY 10016, USA

[6]Ph.D. Programs in Biochemistry, The Graduate Center of the City University of New York, New York, NY 10016, USA

[7]Department of Microbiology, Biochemistry, and Molecular Genetics, New Jersey Medical School, Rutgers Biomedical Health Sciences, Newark, NJ 07103, USA

* Correspondence: junyong.choi@qc.cuny.edu; heesook@njms.rutgers.edu

Supplementary Tables

Supplementary Table 1. EC_{50} values obtained from cell growth experiments comparing the toxicity of JC-229 to *T. brucei*, HEK293, and HeLa cells. All experiments were performed in triplicate. SD=Standard Deviation.

**1-A. Growth inhibition of *T. brucei* and human cell lines by JC-229 (Figure 3f)**				
**Cell lines**	**Best fit EC_{50} (μM)**	**SD**	**R squared**	**Analysis model**
T. brucei	6.54	0.4	0.99	[Inhibitor] vs. norm response, variable slope
HEK293	18.4	2.2	0.94	
HeLa	32.1	2.9	0.89	

**1-B. Growth inhibition of *T. brucei* and human cell lines by JC-229**				
**Cell lines**	**Best fit EC_{50} (μM)**	**SD**	**R squared**	**Analysis model**
T. brucei	6.58*	0.4	0.99	Sigmoidal, 4-PL, X = conc
HEK293	10.3	3.9	0.95	
HeLa	13.2	2.7	0.94	

*: Figure 1f

Supplementary Table 2. K_d values obtained from MST. All experiments were performed in triplicate. SD=Standard Deviation.

2-A. Binding of RPA with ssDNA, dT_{32} (Figure 5, 6, & 7; Supplementary Figure 12)

RPA proteins	Best fit K_d (nM)	SD	R squared	Analysis model
*Tb*RPA1 DBD-AB	24.6	12.4	0.97	Binding; one site, total
*Hs*RPA1 DBD-AB	48.0	37.4	0.96	Binding; one site, total
*Tb*RPA	13.2	3.1	0.90	Binding; one site, total
*Hs*RPA	15.8	6.6	0.97	Binding; one site, total
*Tc*RPA1 DBD-AB	12.6	3.7	0.97	Binding; one site, total
*Lmex*RPA1 DBD-AB	13.2	1.6	0.98	Binding; one site, total
*Tb*RPA1 DBD-AB, S105T	21.9	2.7	0.97	Binding; one site, total
*Tc*RPA1 DBD-AB, T105S	52.4	27.9	0.98	Binding; one site, total
*Tb*RPA1 DBD-AB, F64A	No binding	nd	nd	Binding; one site, total
*Tb*RPA1 DBD-AB, W188A	No binding	nd	nd	Binding; one site, total

2-B. RPA binding with ssDNA in different length (Supplementary Figure 11)

RPA proteins (ssDNA probe)	Best fit K_d (nM)	SD	R squared	Analysis model
*Tb*RPA1 DBD-AB (dT_{32})	24.6	12.4	0.97	Binding; one site, total
*Tb*RPA1 DBD-AB (dT_{20})	30.5	1.1	0.90	Binding; one site, total
*Tb*RPA1 DBD-AB (dT_{12})	60.4	44.3	0.76	Binding; one site, total
*Tb*RPA complex (dT_{32})	15.5	4.3	0.99	Binding; one site, total
*Tb*RPA complex (dT_{20})	17.0	2.1	0.89	Binding; one site, total
*Tb*RPA complex (dT_{12})	256	347	0.84	Binding; one site, total

2-C. ssDNA-binding activity of *Tb*RPA complex containing S105T mutation (Supplementary Figure 18)

RPA proteins (ssDNA probe)	Best fit K_d (nM)	SD	R squared	Analysis model
*Tb*RPA complex (dT_{32})	15.5	4.3	0.99	Binding; one site, total
*Tb*RPA-S105T complex (dT_{32})	18.8	2.0	0.95	Binding; one site, total

2-D. Binding of *Tb*RPA1 DBD-A or DBD-B with ssDNA (Supplementary Figure 13)

RPA1 proteins (ssDNA probe)	Best fit K_d (nM)	SD	R squared	Analysis model
*Tb*RPA1 DBD-A (dT_{32})	5,067	nd	0.53	Binding; one site, total
*Tb*RPA1 DBD-A (dT_{20})	No binding	nd	nd	Binding; one site, total
*Tb*RPA1 DBD-B (dT_{32})	unstable	nd	nd	Binding; one site, total
*Tb*RPA1 DBD-B (dT_{20})	No binding	nd	nd	Binding; one site, total

Supplementary Table 3. IC_{50} values obtained from MST. All experiments were performed in triplicate. SD=Standard Deviation.

**3-A. Inhibition of RPA-ssDNA binding by JC-229 (dT_{32}) (Figure 5, 6, & 7)**				
**DBD-AB**	**Best fit IC_{50} (nM)**	**SD**	**R squared**	**Analysis model**
*Tb*RPA1 DBD-AB	228	11.0	0.95	Sigmoidal, 4-PL, X = conc
*Hs*RPA1 DBD-AB	No inhibition	nd	nd	Sigmoidal, 4-PL, X = conc
*Tb*RPA complex	24.5	2.0	0.96	Sigmoidal, 4-PL, X = conc
*Hs*RPA complex	No inhibition	nd	nd	Sigmoidal, 4-PL, X = conc
*Tc*RPA1 DBD-AB	No inhibition	nd	nd	Sigmoidal, 4-PL, X = conc
*Lmex*RPA1 DBD-AB	No inhibition	nd	nd	Sigmoidal, 4-PL, X = conc
*Tb*RPA1 DBD-AB, S105T	No inhibition	nd	nd	Sigmoidal, 4-PL, X = conc
*Tc*RPA1 DBD-AB, T105S	159×10^3	nd	0.87	Sigmoidal, 4-PL, X = conc

**3-B. Inhibition of *Tb*RPA binding with ssDNA in different length (Supplementary Figure 11)**				
**RPA proteins (ssDNA probe)**	**Best fit IC_{50} (nM)**	**SD**	**R squared**	**Analysis model**
*Tb*RPA1 DBD-AB (dT_{32})	228	11.0	0.95	Sigmoidal, 4-PL, X = conc
*Tb*RPA1 DBD-AB (dT_{20})	881	234	0.91	Sigmoidal, 4-PL, X = conc
*Tb*RPA1 DBD-AB (dT_{12})	2,799	459	0.72	Sigmoidal, 4-PL, X = conc
*Tb*RPA complex (dT_{32})	33.9	13	0.91	Sigmoidal, 4-PL, X = conc
*Tb*RPA complex (dT_{20})	33.9	20	0.72	Sigmoidal, 4-PL, X = conc
*Tb*RPA complex (dT_{12})	nd	nd	nd	nd

**3-C. Inhibition of ssDNA-binding activity of S105T *Tb*RPA complex by JC-229 (Supplementary Figure 18)**				
**RPA proteins (ssDNA probe)**	**Best fit IC_{50} (nM)**	**SD**	**R squared**	**Analysis model**
*Tb*RPA complex (dT_{32})	33.9	13	0.91	Sigmoidal, 4-PL, X = conc
*Tb*RPA-S105T complex (dT_{32})	48,800	nd	0.25	[Inhibitor] vs. response, variable slope (four parameters)

Supplementary Table 4. K_d and IC_{50} values for *Hs*DBD-F and Cy5-ATRIP interaction and inhibition by JC-229 (by MST). All experiments were performed in triplicate. SD=Standard Deviation.

**4-A. Binding of *Hs*DBD-F with ATRIP peptide (Supplementary Figure 14)**				
**Proteins**	**Best fit K_d (nM)**	**SD**	**R squared**	**Analysis model**
*Hs*DBD-F	34.3 x10^3	3.8 x10^3	0.95	Binding; one site, total
BSA	nd	nd	0.25	Binding; one site, total

**4-B. Inhibition of *Hs*DBD-F and ATRIP interaction by JC-229 (Supplementary Figure 14)**				
**RPA proteins**	**Best fit IC_{50} (nM)**	**SD**	**R squared**	**Analysis model**
*Hs*DBD-F	60.8 x10^3	44.6 x10^3	0.94	Sigmoidal, 4-PL, X = conc

Supplementary Table 5. EC_{50} values obtained from cell growth experiments comparing the toxicity of JC-229 to WT, *Tb*RPA1 sKO, and *Tb*RPA1 S105T mutant strains. All experiments were performed in triplicate. SD=Standard Deviation.

**5-A. Growth inhibition of WT and S105T mutant *T. brucei* cells by JC-229 (Figure 8)**				
***T. brucei* cell lines**	**Best fit EC_{50} (μM)**	**SD**	**R squared**	**Analysis model**
WT (WT / WT)	5.68	0.16	0.98	[Inhibitor] vs. norm response, variable slope
Single KO (Δ / WT)	4.93	0.15	0.98	
S105T clone 1 (Δ / RPA1-S105T)	30.4	1.37	0.94	
S105T clone 2 (Δ / RPA1-S105T)	21.7	2.36	0.94	
S105T clone 3 (Δ / RPA1-S105T)	19.6	5.77	0.89	

**5-B. Growth inhibition of WT and S105T mutant *T. brucei* cells by JC-229**				
***T. brucei* cell lines**	**Best fit EC_{50} (μM)**	**SD**	**R squared**	**Analysis model**
WT (WT / WT)	5.65	0.22	0.98	Sigmoidal, 4-PL, X = conc
Single KO (Δ / WT)	4.61	0.17	0.99	
S105T clone 1 (Δ / RPA1-S105T)	17.4	24.3	0.94	
S105T clone 2 (Δ / RPA1-S105T)	30	31.9	0.94	
S105T clone 3 (Δ / RPA1-S105T)	16.5	5.3	0.89	

Supplementary Table 6. *T. brucei* cell lines used in this study.

Name	Genotype	Reference
2T1	PUR at rDNA locus (landing pad), T7 RNA Polymerase & Tet Repressor with PHLEO marker	[1]
AMT2	PHLEO marker in 2T1 strain was replaced with NEO by transfecting pT7-NEO vector	This study
AMT5	*Tb*RPA1 RNAi vector (pOH10) integrated at rDNA locus in AMT2 WT strain; KD clone 1	This study
AMT6	*Tb*RPA1 RNAi vector (pOH10) integrated at rDNA locus in AMT2 WT strain; KD clone 2	This study
HSTB-904	WT in Single marker (SM) background, the active BES1 promoter marked with BSD, Cre-recombinase under Tet control at rDNA spacer	[2,3]
AMT30	Single KO (WT/*Tb*RPA1Δ): pSUN18 (a *Tb*RPA1 KO vector with HYG-TK) transfected in HSTB-904 to delete one allele of *Tb*RPA1	This study
AMT38	*Tb*RPA1-S105T mutant clone 1 (S105T/Δ): AMT30 strain transfected with pAD10 (*Tb*RPA1-S105T knock-in vector with PUR marker)	This study
AMT39	*Tb*RPA1-S105T mutant clone 2 (S105T/Δ): AMT30 strain transfected with pAD10 (*Tb*RPA1-S105T knock-in vector with PUR marker)	This study
AMT41	*Tb*RPA1-S105T mutant clone 3 (S105T/Δ): AMT30 strain transfected with pAD10 (*Tb*RPA1-S105T knock-in vector with PUR marker)	This study

Supplementary Table 7. Plasmids used in this study.

Name	Description	Reference
p2T7-NEO	T7 RNA polymerase, NEO marker	This study
pOH10	*Tb*RPA1 RNAi in p2T7-TA vector	This study
pSR2	T7-lacO-rbs-*Tb*RPA2 in pGEM vector	This study
pSR3	T7-lacO-rbs-*Tb*RPA3 in pGEM vector	This study
pSR5	6xHis-*Tb*RPA1 DBD-AB in pET28a vector	This study
pSR6	*Tb*RPA3 in pET28a vector	This study
pSR7	*Tb*RPA2 and *Tb*RPA3 in pET28a vector	This study
pSR8	6xHis-*Tb*RPA1, *Tb*RPA2, *Tb*RPA3 in pET28a (all three subunits have their own T7 promoter-lacO-rbs)	This study
pSR9	6xHis-*Tb*RPA1 DBD-AB-F64A in pET28a vector	This study
pSR10	6xHis-*Tb*RPA1 DBD-AB-W188A in pET28a vector	This study
pAM.Tc1	6xHis-*Tc*RPA1 DBD-AB in pET28a	This study
pSR15	6xHis-*Hs*RPA1 DBD-AB in pET28a	This study
pAM.Lm1	6xHis-*Lmex*RPA1 DBD-AB in pET28a	This study
pSR18i	6xHis-*Hs*RPA1, *Hs*RPA2, *Hs*RPA3 in pET28a (all three subunits have their own T7 promoter-lacO-rbs)	This study
pSR21	6xHis-*Tb*RPA1 DBD-AB with S105T mutation in pET28a	This study
pSR23	6xHis-*Tc*RPA1 DBD-AB with T105S mutation in pET28a	This study
p11d-tRPA(123)	*Hs*RPA1, *Hs*RPA2, *Hs*RPA3 in pET11d	[4]
pSR26	6xHis-*Tb*RPA complex with *Tb*RPA1-S105T mutation in pET28a vector	This study
pSR28	6xHis-*Tb*RPA1 DBD-A in pET28a vector	This study
pSR29	6xHis-*Tb*RPA1 DBD-B in pET28a vector	This study
pSR31	6xHis-*Hs*RPA1 DBD-F in pET28a vector	This study
pSUN18	*Tb*RPA1 deletion vector with floxed HYG-TK marker	This study
pAD10	*Tb*RPA1-S105T knock-in vector with PUR marker	This study

Supplementary Table 8. Oligonucleotides used in this study.

Name	Description	Sequences
778	Forward to amplify *Tb*RPA1 RNAi target region	ATGCAGCAGCCATCACAACAACAG
779	Reverse to amplify *Tb*RPA1 RNAi target region	TCACGTCAGGGATGTTTGGATTAA
1089	Forward to amplify T7-lacO-rbs-*Tb*RPA3 (HindIII)	tttaagcttcgaaattaatacgactcactataggggaattgtgagcggataacaattccccttttaagaaggagtaccCGTATGCGTTCAGCTGTTAAT
1090	Reverse to amplify T7-lacO-rbs-*Tb*RPA3 (XhoI)	tttctcgagCTAAAAGAGTGCTGGAAACTT
1091	Forward to amplify T7-lacO-rbs-*Tb*RPA2 (BamHI)	tttggatcccgaaattaatacgactcactataggggaattgtgagcggataacaattccccttttaagaaggagatataccATGGAAGGAAGTGGAAGTA
1092	Reverse to amplify T7-lacO-rbs-*Tb*RPA2 (HindIII)	tttaagcttTTAAATGCCAAACTTACAATC
1066	Forward to amplify and clone *Tb*RPA1 full-length or DBD-AB into pET28a (NheI)	tttgctagcCAGCAGCCATCACAACAACAGA
1093	Reverse to amplify full-length*Tb*RPA1 with BglII (PCR with forward oligo 1066)	tttagatctTTACAAGTAGGCATTAATGCAT
1094	Reverse to amplify *Tb*RPA1 DBD-AB with stop codon, XhoI (PCR with forward oligo 1066)	tttctcgagTTATCCCTGCATTGAAAGTGAACT
1221	Forward to introduce *Tb*RPA1-F64A mutation by Quick change	ATCCGAGCGACGGTA**gc**CAATGAGGCCGTCGAC
1222	Reverse to introduce *Tb*RPA1-F64A mutation by Quick change	AGAAGCAGACTCGTCAATGAGCGT
1223	Forward to introduce *Tb*RPA1-W188A mutation by Quick change	ATTGACGTCACCCTG**gc**GAACGAAAACGCCAAA
1224	Reverse to introduce *Tb*RPA1-W188A mutation by Quick change	CCCCGCGGTGGAATCGGCAAC
1225	Forward to amplify *Tc*RPA1 DBD-AB (NheI)	tttgctagcCAACACCCGAGCAATCAGCA
1226	Reverse to amplify *Tc*RPA1 DBD-AB (stop codon, HindIII)	tttaagcttTTAACCCAAGGAAGAGAGCGAAGA
1227	Forward to amplify *Hs*RPA1 DBD-AB (NheI)	tttgctagcCAGTCCAAAGTGGTGCCCATT
1228	Reverse to amplify *Hs*RPA1 DBD-AB (stop codon, BamHI)	tttggatccTTAGATGGAAACACCATCTAAGGC
1232	Reverse to amplify upstream region of *Hs*RPA1 to tag the N-terminus with 6xHis in p11d-tRPA-123 vector	GCTAGCCATATGGCTGCCGCGCGGCACCAGGCCGCTGCTGTGATGATGATGATGATGGCTGCTGCCCATGGTATATCTCCTTCTTAAAGTTAA
1233	Forward to amplify downstream region of *Hs*RPA1 to tag the N-terminus with 6xHis in p11d-tRPA-123 vector	ATGGGCAGCAGCCATCATCATCATCATCACAGCAGCGGCCTGGTGCCGCGCGGCAGCCATATGGCTAGCATGGTCGGCCAGCTGAGCGAG

1256	Forward to introduce 6xHis tag to *Hs*RPA1 (PCR with reverse oligo 1232)	GATCTCGACGCTCTCCCTTATGCG
1257	Reverse to introduce 6xHis tag to *Hs*RPA1 (PCR with forward oligo 1233)	CTTCATAGCTCTTGCAGATCCCGA
AM75	Forward to introduce S105T mutation in *Tb*RPA1 DBD-AB by Quick change	AATGACTACGAGTTGA**c**CTTTGACAACACCTGT
AM76	Reverse to introduce S105T mutation in *Tb*RPA1 DBD-AB by Quick change	ATTCACATTACTGAACTTACGGTTGG
AM79	Forward to introduce T105S mutation in *Tc*RPA1 DBD-AB by Quick change	AACGACTATGAGCTG**t**CGTTTGATAGCTCGAGT
AM80	Reverse to introduce T105S mutation in *Tc*RPA1 DBD-AB by Quick change	GTTCACGTTGCTGAACTTGCGATTT
Lmex Fw	Forward to amplify *Lmex*RPA1 DBD-AB (NheI)	tttgctagcCAACAGCCGGGCAGCCACCAG
Lmex Rv	Reverse to amplify *Lmex*RPA1 DBD-AB (Stop codon, BamHI)	tttggatccCCCCTGCGACGACAAGGACA
1290	Reverse to amplify *Tb*RPA1 DBD-A with stop codon, XhoI (PCR with forward oligo 1066)	tttctcgagTTAAATCGACGAGCTAACGACATC
1291	Forward to amplify *Tb*RPA1 DBD-B and clone into pET28a (NheI site and start codon)	tttgctagcATGTCGATTCCCCTACAGCGGTAT
1295	Forward to amplify *Hs*RPA1 DBD-F & clone in pET28a (NheI)	tttgctagcATGGTCGGCCAGCTGAGCGAG
1296	Reverse to amplify *Hs*RPA1 DBD-F & clone in pET28a (stop codon, XhoI)	tttctcgagTTACTGTGTTCCCCCAGAAGTGTG
162	Forward to genotype *Tb*RPA1 KO-HYG-TK allele; anneals on TK gene	CTGCAACTTACCTCCGGGAT
776	Forward to genotype *Tb*RPA1 alleles, including WT, KO-HYG-TK, and S105T mutant	GGATTTAACCAACAAGAGAGAAAGAACA
778	Forward to amplify *Tb*RPA1 WT and S105T alleles for Sanger sequencing (PCR with reverse oligo 1299)	ATGCAGCAGCCATCACAACAACAG
1299	Reverse to genotype *Tb*RPA1 alleles, including WT, KO-HYG-TK, and S105T mutant	TTCACGCGAAATATCAGTCGTGTC
AM88	Forward to amplify TUB-intergenic region and PUR from pMOTag2H vector (with SpeI)	actagtTACCCTTACGATGTGCCT
AM89	Reverse to amplify TUB-intergenic region and PUR from pMOTag2H vector (with BamHI)	ggatccTCAGGCACCGGG
AM90	Forward to amplify *Tb*RPA1-S105T CDS from pSR26 & clone into pSUN18 to make KI vector (PsiI & ATG)	ttataaATGCAGCAGCCATCACAA
AM91	Reverse to amplify *Tb*RPA1-S105T CDS from pSR26 & clone into pSUN18 to make KI vector (SpeI & stop codon)	actagtTTATTACAAGTAGGCATTAAT

Supplementary Figures

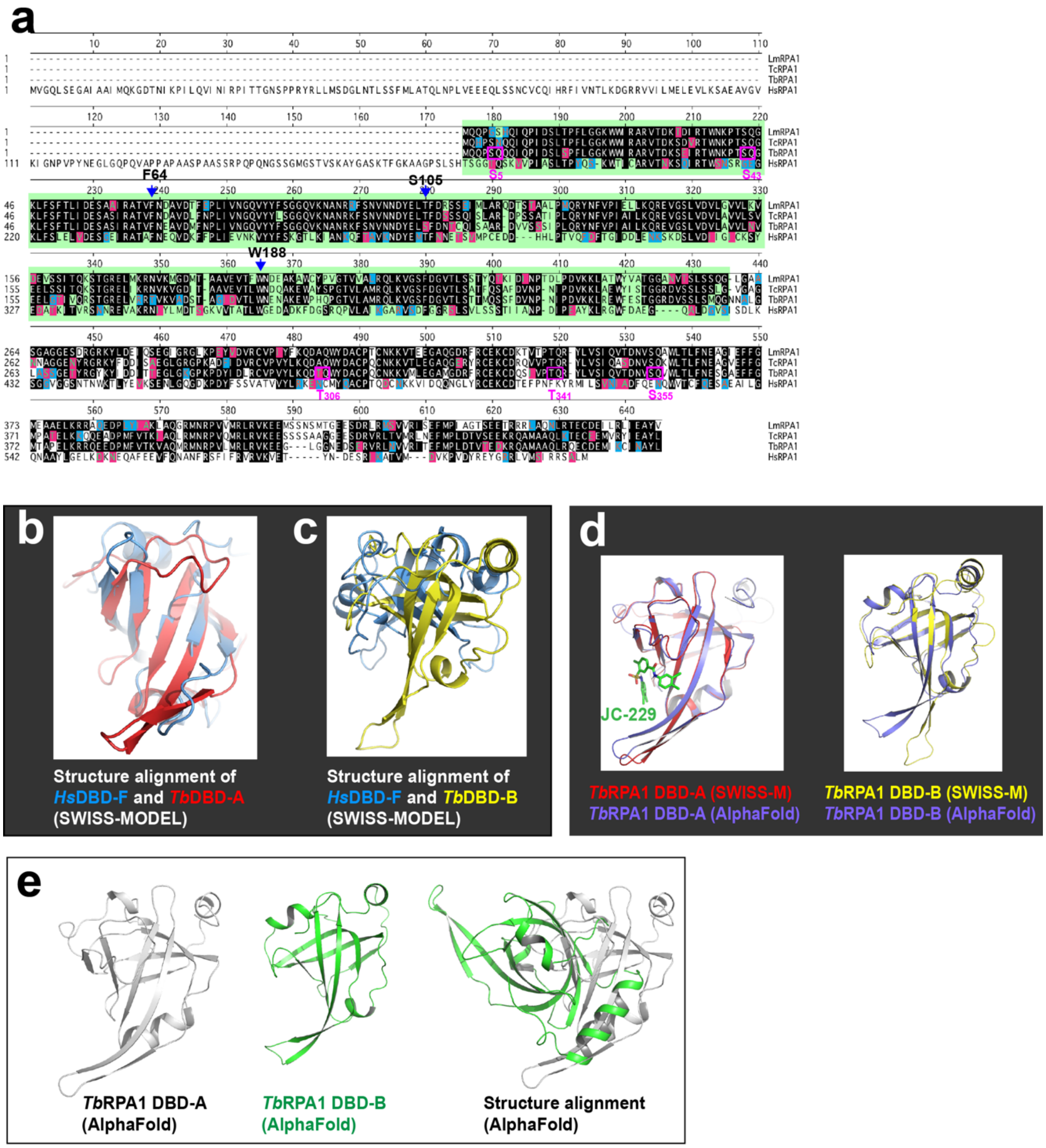

Supplementary Figure 1. Sequence alignment of *L. major* (same as *L. mexicana*), *T. cruzi*, *T. brucei* and human RPA1 and structure comparison by SWISS vs. AlphaFold models. a Sequence alignment of RPA1. DNA-Binding Domain A and B (DBD-AB) are highlighted in green. Three of important amino acid residues indicated with blue arrows were characterized by site-directed mutagenesis, EMSA, and MST assays (F64A and W188A in Supplementary Fig. 12; S105T in Fig. 7 and Supplementary Fig. 17, 18). Serine or Threonine residues in SQ/TQ motifs are labeled and indicated in pink box and pink font. **b, c** Comparison of *Hs*RPA1 DBD-F with *Tb*RPA1 DBD-A (b; also shown in Fig. 1b) and *Tb*RPA1 DBD-B (c) obtained from SWISS-MODEL. **d** Comparison of *Tb*RPA1 DBD-A (red) and DBD-B (yellow) 3-D structures obtained from SWISS-MODEL and AlphaFold (purple). Each domain was aligned separately by using Pymol software. JC-229 is shown in green. **e** The 3-D structure models of *Tb*RPA1 DBD-A (*left*) and *Tb*RPA1 DBD-B (*middle*) generated from AlphaFold and their structure alignment by Pymol software (*right*).

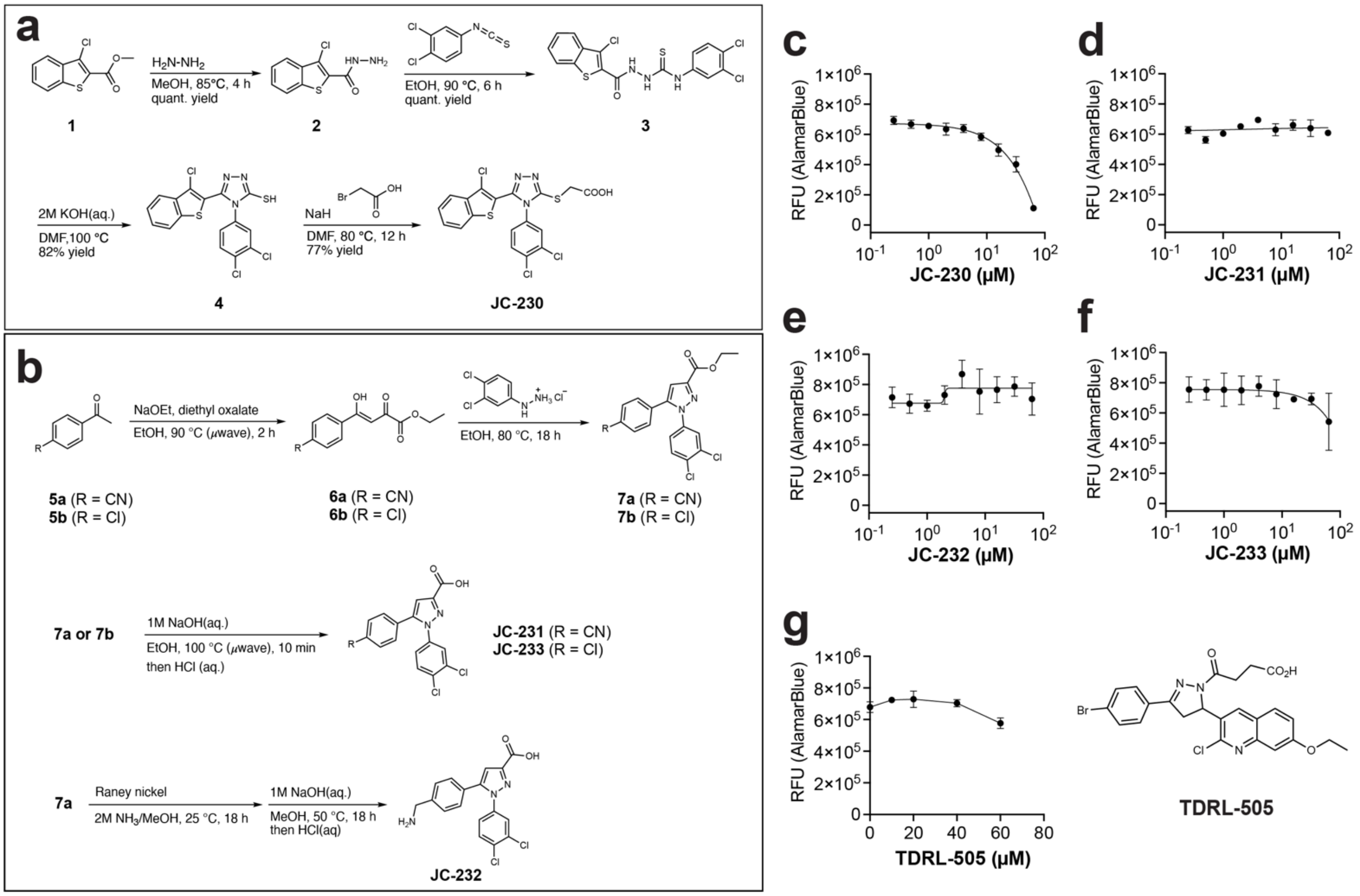

Supplementary Figure 2. Inhibition of *T. brucei* cell proliferation by JC compounds and TDRL-505 tested at the screening stage. a Synthesis scheme for JC-230. **b** Synthesis scheme for JC-231, 232, and 233. **c-f** Dose-dependent growth inhibition of *T. brucei* by JC-230, JC-231, JC-232 and JC-233. *T. brucei* cells were treated with increasing concentrations of JC-compounds (0.25 to 64 μM) for 72 hours and viability was determined with the AlamarBlue assay. **g** Dose-dependent growth inhibition of *T. brucei* by TDRL-505. *T. brucei* cells were treated with increasing concentrations of TDRL-505 (10 to 60 μM) for 72 hours and viability was determined with the AlamarBlue assay. Three biological replicates were used for each measurement for Supplementary Fig. 2c-g (n=3). Error bars indicate mean ±SD. A standard 4-Parameter Logistic (4-PL) curve from GraphPad Prism software generated the EC_{50} values for JC-230 (EC_{50}, 50μM). Source data are provided as a Source Data file.

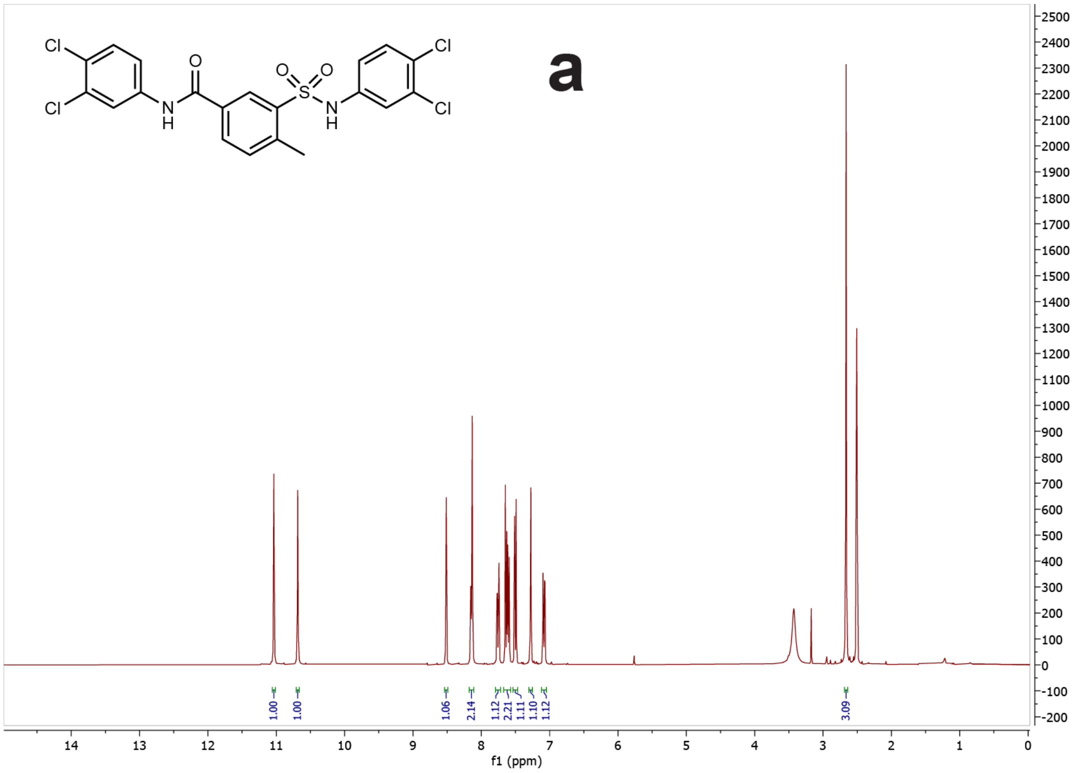

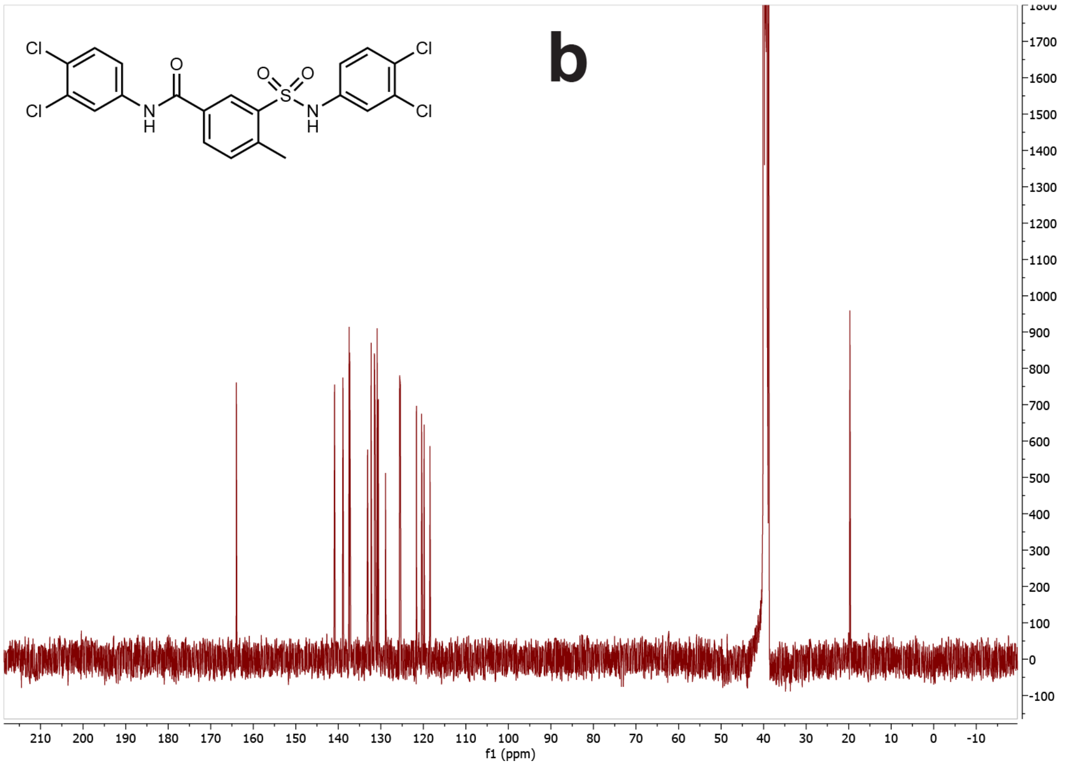

Supplementary Figure 3. NMR Spectroscopic analysis of JC-229. a ^{1}H NMR spectrum of JC-229. **b** ^{13}C NMR spectrum of JC-229. Source data are provided as a Source Data file.

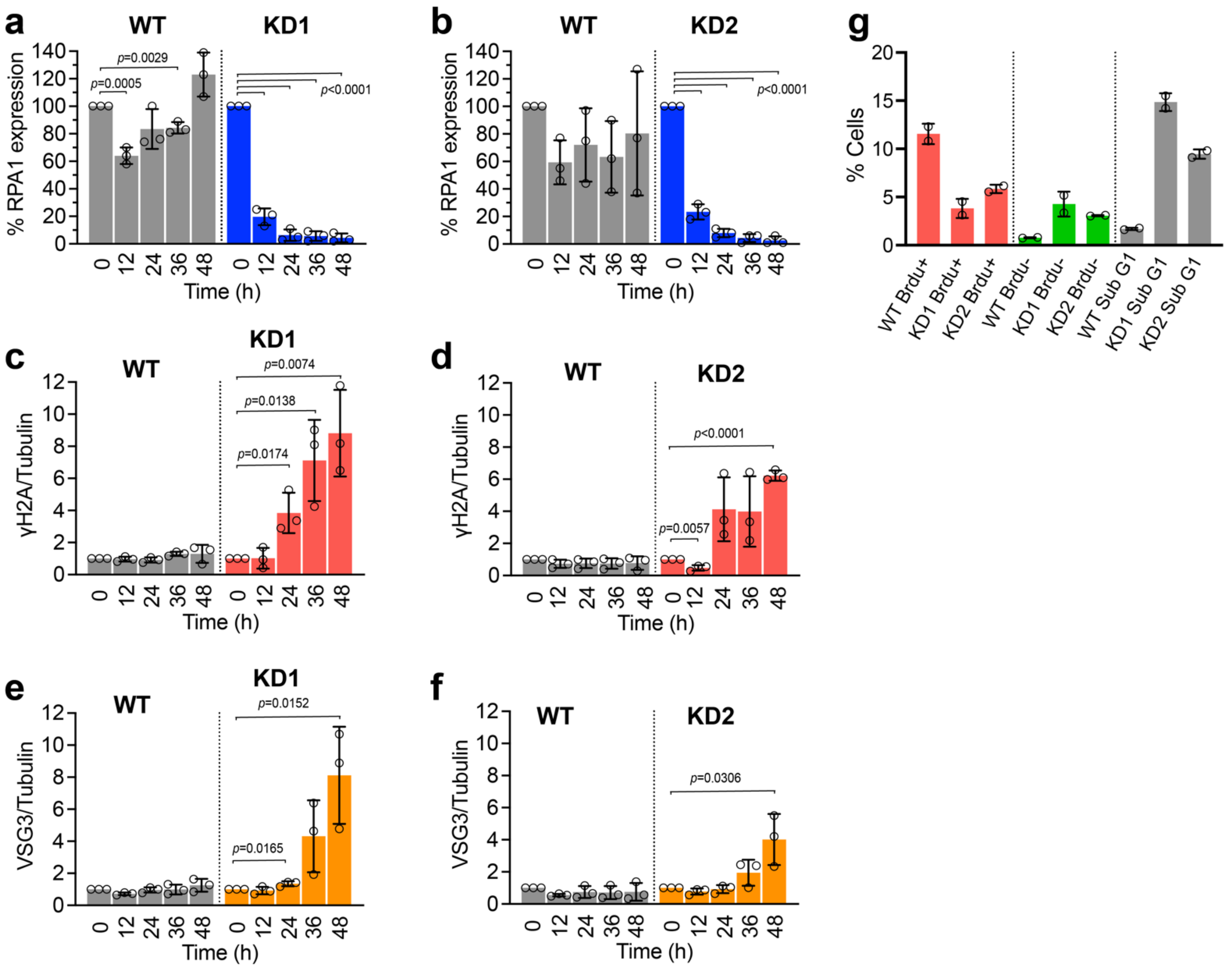

Supplementary Figure 4. Quantification of protein levels in immunoblots (Fig. 2c) and quantification of BrdU-stained cells (Fig. 2e). a, b % *Tb*RPA1 protein level in *Tb*RPA1 KD1 and KD2 cells: Signal intensities of *Tb*RPA1 bands were quantified using ImageJ software and normalized to those of Tubulin protein bands and fold change was obtained by normalizing to 0 hr time point. **c-f** Fold change of γH2A (c, d) and VSG3 (e, f): γH2A and VSG3 bands were analyzed as RPA1 bands and fold changes are plotted. Blots from three independent experiments were quantified for Supplementary Fig. 4a-f (n=3). Error bars indicate mean ±SD. An unpaired two-sided Student's t-test was performed. **g** Quantification of BrdU positive (+), BrdU negative (-) and sub-G1 populations in WT, KD1 and KD2 at 24-hour post-RNAi induction. Two independent flow experiments were averaged (n=2). Error bars indicate mean ±SD. Source data are provided as a Source Data file.

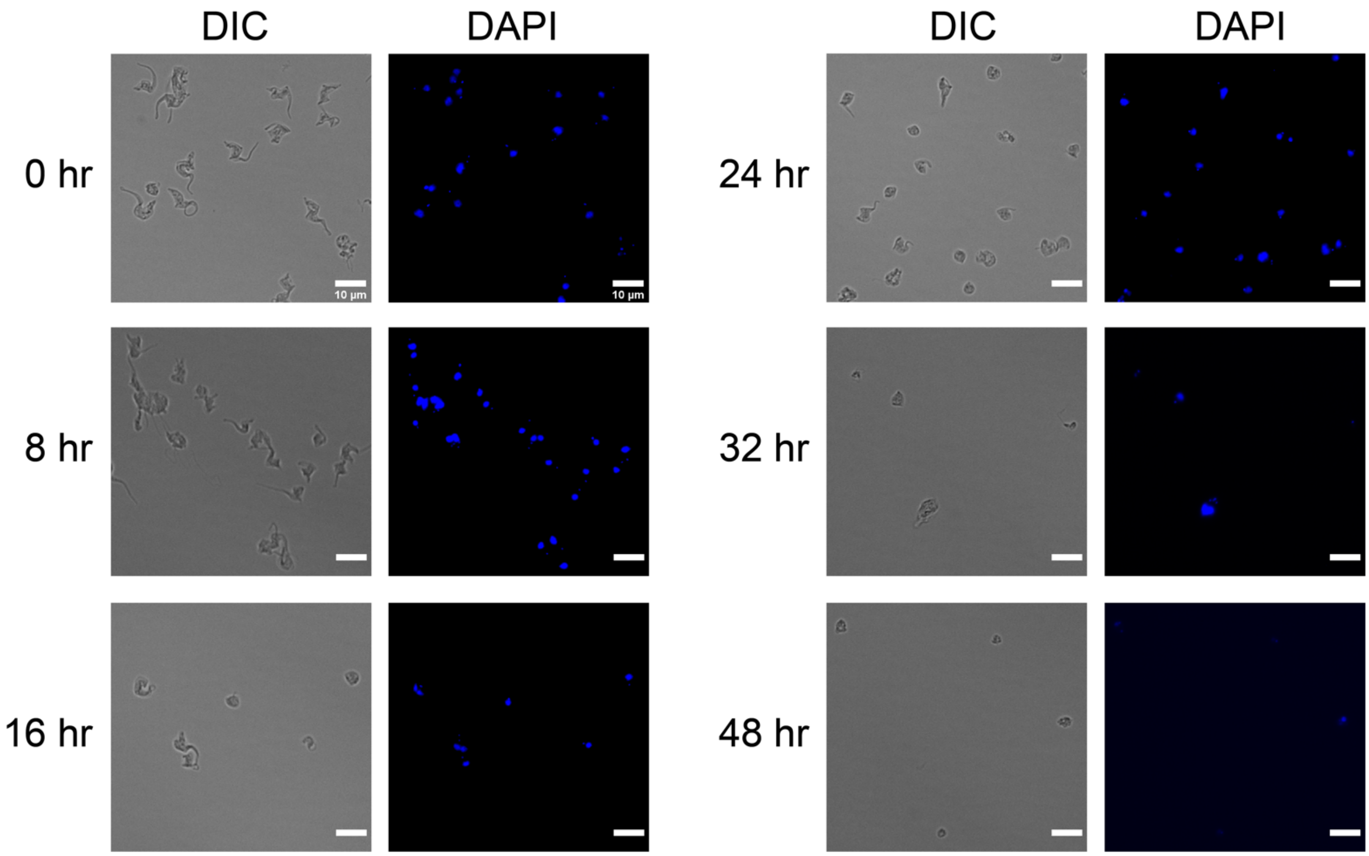

Supplementary Figure 5. Microscopic analysis of JC-229 treated *T. brucei* cells. Cells collected at indicated time points were fixed in 1% paraformaldehyde for 10 min and stained with DAPI. Images were obtained from EVOS M5000 microscope. Scale bar, 10 μm. Twenty images were taken for each timepoint with similar results. Source data are provided as a Source Data file.

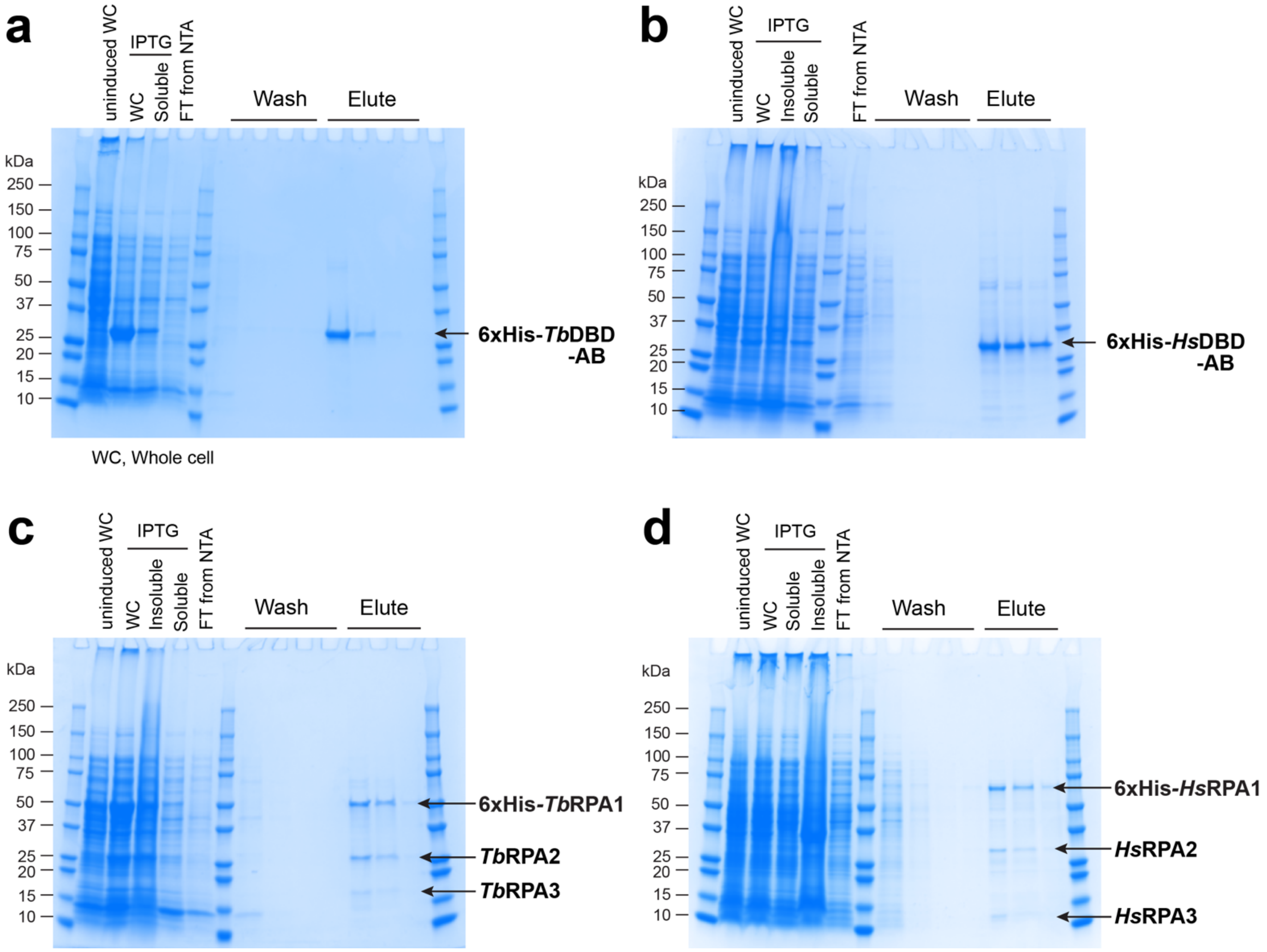

Supplementary Figure 6. Ni-NTA purification of *Tb*RPA1 DBD-AB (a), *Hs*RPA1 DBD-AB (b), *Tb*RPA complex (c) and *Hs*RPA complex (d) from *E. coli*. Representative SDS-PAGE gels are shown. WC= whole cell, FT= flow through. Three independent purifications were performed for Supplementary Fig. 6a-d with similar results. Source data are provided as a Source Data file.

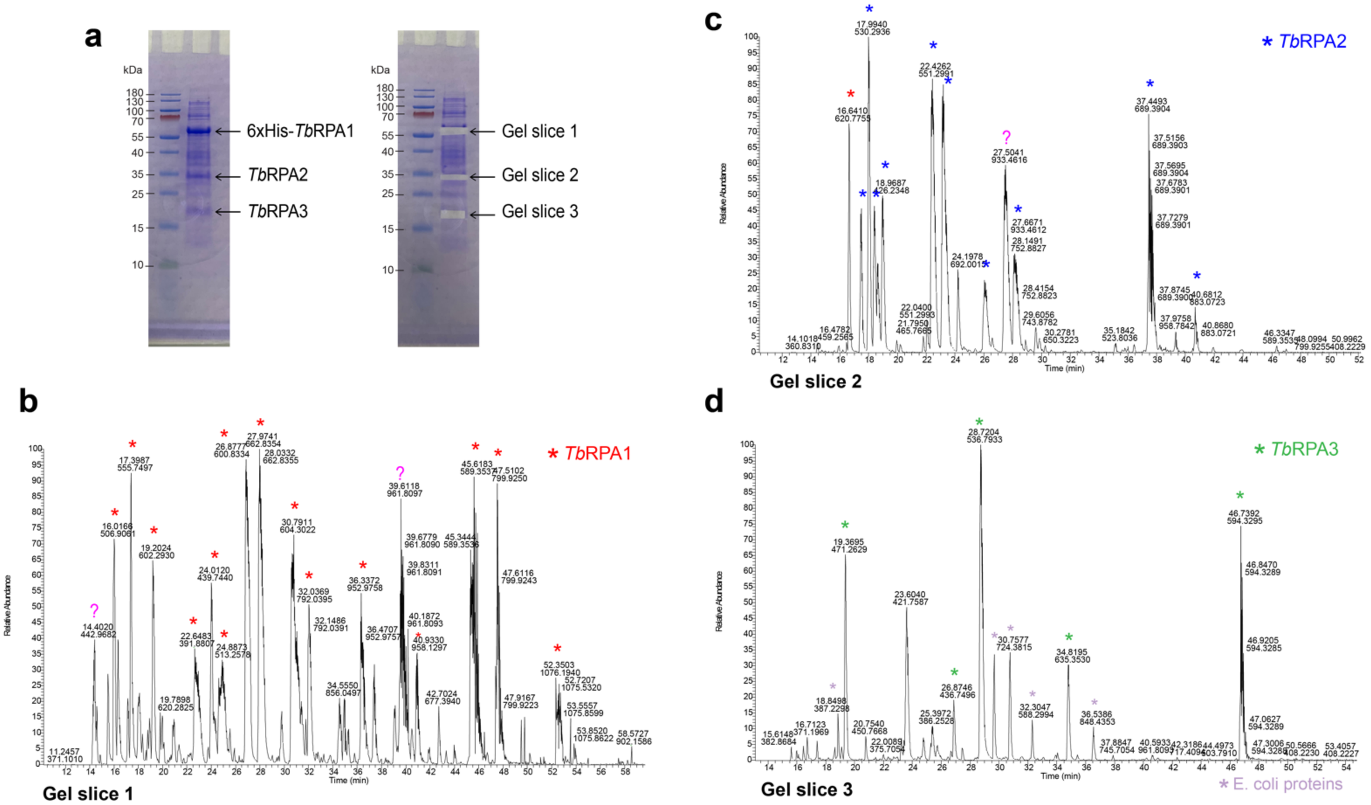

Supplementary Figure 7. LC-MS/MS of purified *Tb*RPA complex. **a** SDS-PAGE blots showing purified *Tb*RPA complex. 6xHis-*Tb*RPA1, *Tb*RPA2, and *Tb*RPA3 subunits are indicated with arrows. Gel slices containing these proteins were analyzed by mass spectrometry. **b-d** LC-MS/MS data analysis. A representative nano LC-MS/MS base peak chromatogram, showing the detection of signature peptides for *Tb*RPA1, *Tb*RPA2 and *Tb*RPA3 (shown in red, blue, and green asterisks, respectively). Source data are provided as a Source Data file.

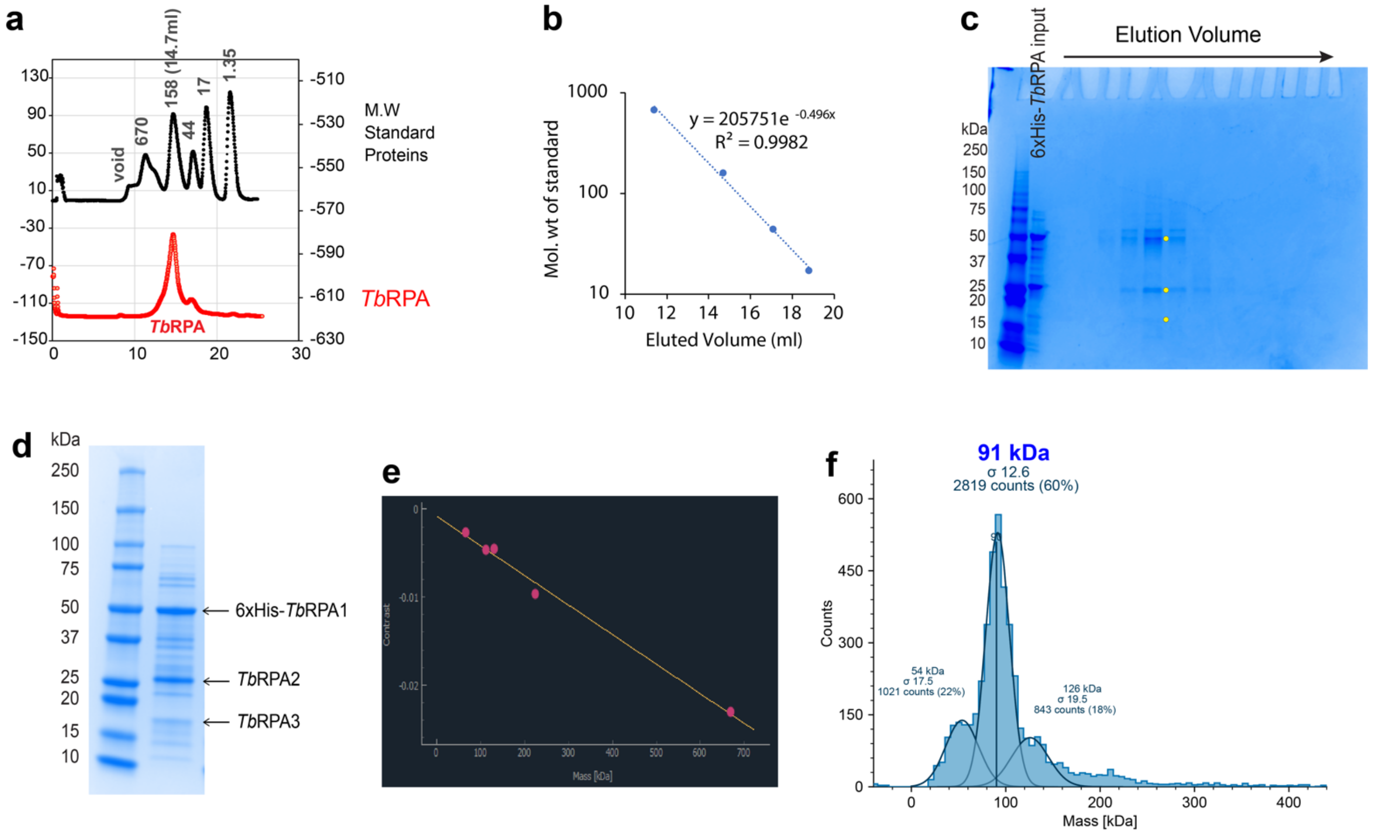

Supplementary Figure 8. Size exclusion chromatography (SEC) and mass photometry (MP) of purified *Tb*RPA complex. **a-c** Size exclusion chromatography. **a** Purified *Tb*RPA complex was loaded onto a Superdex 200 10/300 column using ÄKTA chromatography system. SEC shows a major peak at an estimated molecular weight of 142 kDa (shown in red). Gel filtration calibration standard containing thyroglobulin (670 kDa), bovine γ globulin (158 kDa), chicken ovalbumin (44 kDa), equine myoglobin (17 kDa), and vitamin B12 (1.35 kDa) was separated using the same column to estimate the size of *Tb*RPA complex. **b** A standard curve generated from protein markers using their eluted volume. **c** SDS-PAGE gel analysis of eluted fractions of *Tb*RPA sample from SEC. The experiment was performed two times with similar results. **d-f** Mass photometry. **d** SDS-PAGE blot showing purified *Tb*RPA complex used for MP analysis. Three independent experiments were performed with similar results. **e** A calibration curve generated from molecular standards. **f** Histogram of population distribution of purified *Tb*RPA. The molecular weight of *Tb*RPA is estimated to be ~91 kDa from the calibration curve in e. Source data are provided as a Source Data file.

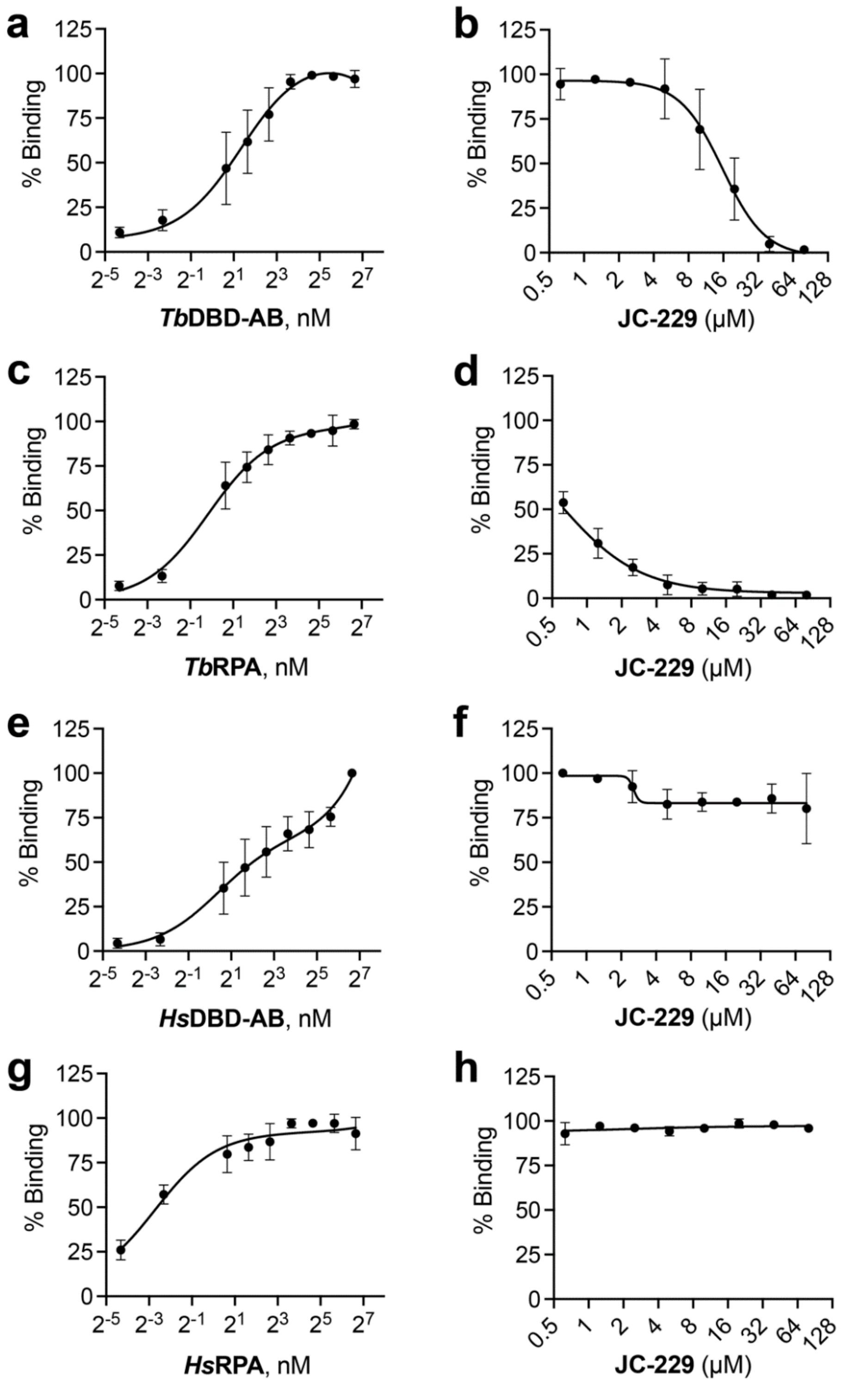

Supplementary Figure 9. Quantification of RPA-ssDNA complexes on EMSA gels in Fig. 4. Levels of protein-ssDNA complexes in EMSA gels were quantified using ImageJ software and plotted. **a, b** *Tb*RPA1 DBD-AB (a: ssDNA binding, b: inhibition by JC-229). **c, d** *Tb*RPA complex (c: ssDNA binding, d: inhibition by JC-229). **e, f** *Hs*RPA1 DBD-AB (e: ssDNA binding, f: inhibition by JC-229). **g, h** *Hs*RPA complex (g: ssDNA binding, h: inhibition by JC-229). EMSA gels from three independent experiments were quantified (n=3) by using ImageJ. Error bars indicate mean ±SD. Source data are provided as a Source Data file.

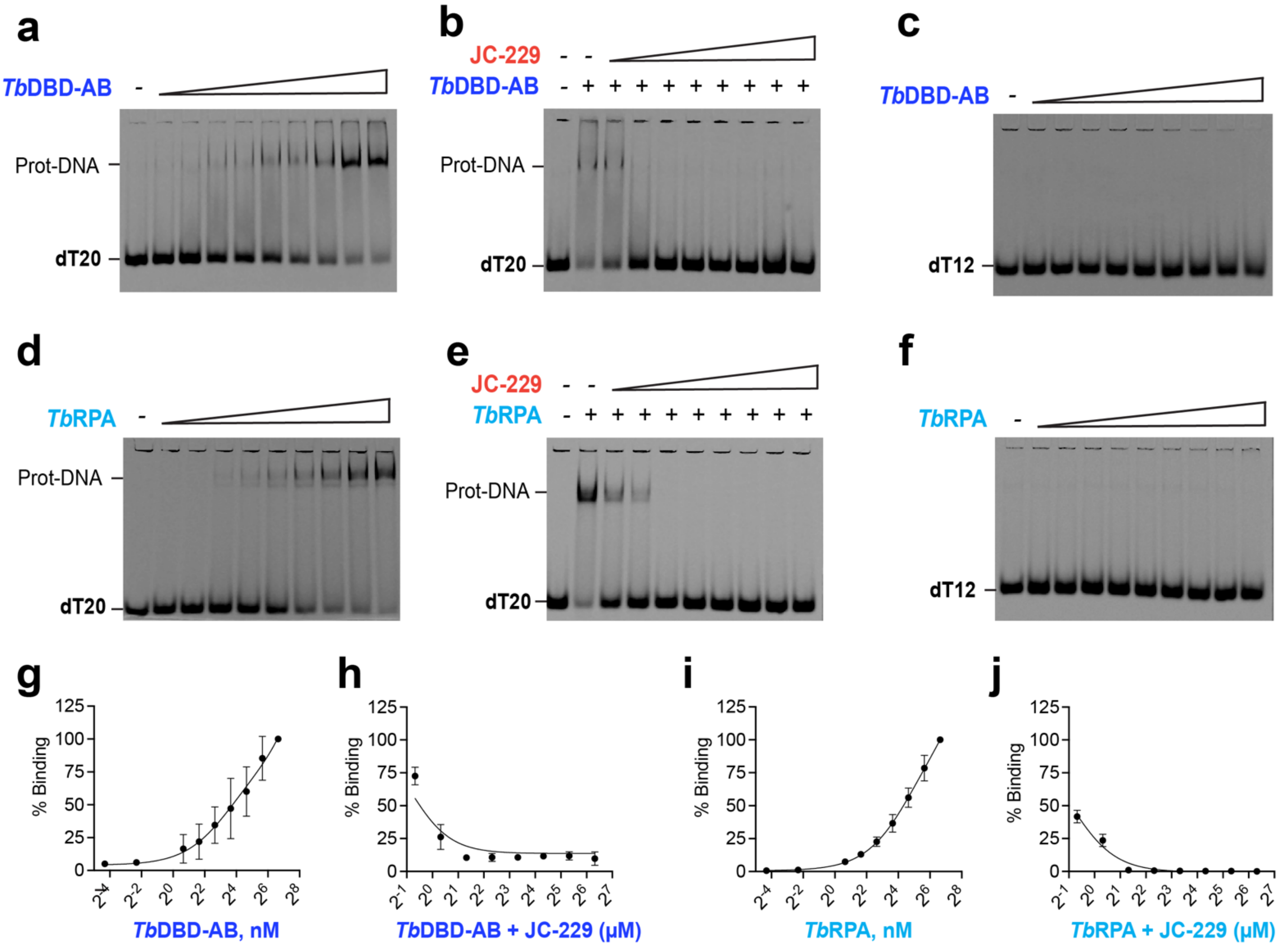

Supplementary Figure 10. ssDNA-binding activities of *Tb*RPA1 DBD-AB and *Tb*RPA complex with shorter ssDNA substrates and JC-229 inhibition (by EMSA). a Binding of *Tb*RPA1 DBD-AB with dT_{20}. Serial dilutions (0.05, 0.2, 1.6, 3.2, 6.25, 12.5, 25, 50 and 100 nM) of *Tb*RPA1 DBD-AB were incubated with 2 nM 5´IRDye800-dT_{20} and samples were analyzed by EMSA. **b** Inhibition of *Tb*DBD-AB's interaction with dT_{20} by JC-229. Serial dilutions (0.625, 1.25, 2.5, 5, 10, 20, 40 and 80 μM) of JC-229 were pre-incubated with 12.5 nM *Tb*RPA1 DBD-AB protein, then incubated with 5´IRDye800-dT_{20}. Inhibition of binding was visualized by EMSA. **c** Binding of *Tb*RPA1 DBD-AB with dT_{12}. **d** Binding of *Tb*RPA complex with dT_{20}. **e** Inhibition of *Tb*RPA complex's interaction with dT_{20} by JC-229. **f** Binding of *Tb*RPA complex with dT_{12}. The ssDNA-binding assay was performed as in a and the inhibition assay as in b. **g-j** Quantification of protein-ssDNA complexes in EMSA gels (a, b, d, and e, respectively) using ImageJ software. EMSA gels from three independent experiments were quantified (n=3) by using ImageJ. Error bars indicate mean ±SD. Source data are provided as a Source Data file.

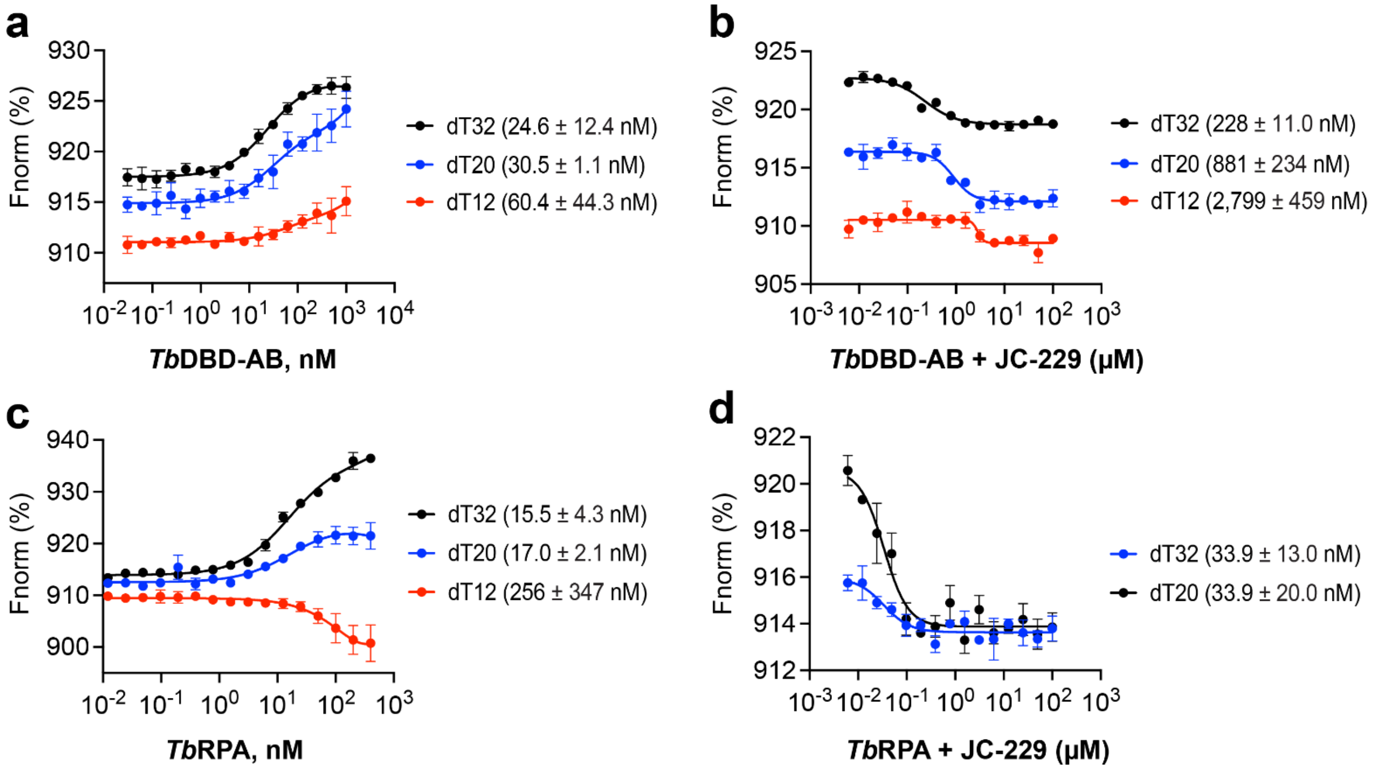

Supplementary Figure 11. ssDNA-binding activities of *Tb*RPA1 DBD-AB and *Tb*RPA complex with shorter ssDNA substrates and JC-229 inhibition (by MST). The MST assay was performed as described in Fig. 5. **a** Comparison of ssDNA-binding activities of *Tb*RPA1 DBD-AB with oligo probes of different lengths, dT_{32}, dT_{20}, or dT_{12}. **b** Comparison of JC-229 inhibition on *Tb*RPA1 DBD-AB's ssDNA binding with dT_{32}, dT_{20}, or dT_{12}. 30 nM of purified protein was used. **c** Comparison of ssDNA-binding activities of *Tb*RPA complex with oligo probes of different lengths, dT_{32}, dT_{20}, or dT_{12}. **d** Comparison of JC-229 inhibition on *Tb*RPA complex's ssDNA binding with dT_{32} or dT_{20}. 25 nM of purified complex was used. Binding with dT_{32}, dT_{20} and dT_{12} are shown in black, blue, and red respectively. Three independent MST experiments were performed (n=3). Error bars indicate mean ±SD. Statistical analysis and plotting were performed with GraphPad Prism software. K_d and IC_{50} values were obtained with a one site binding total and standard 4-PL curve respectively. Source Data are provided as a Source Data file.

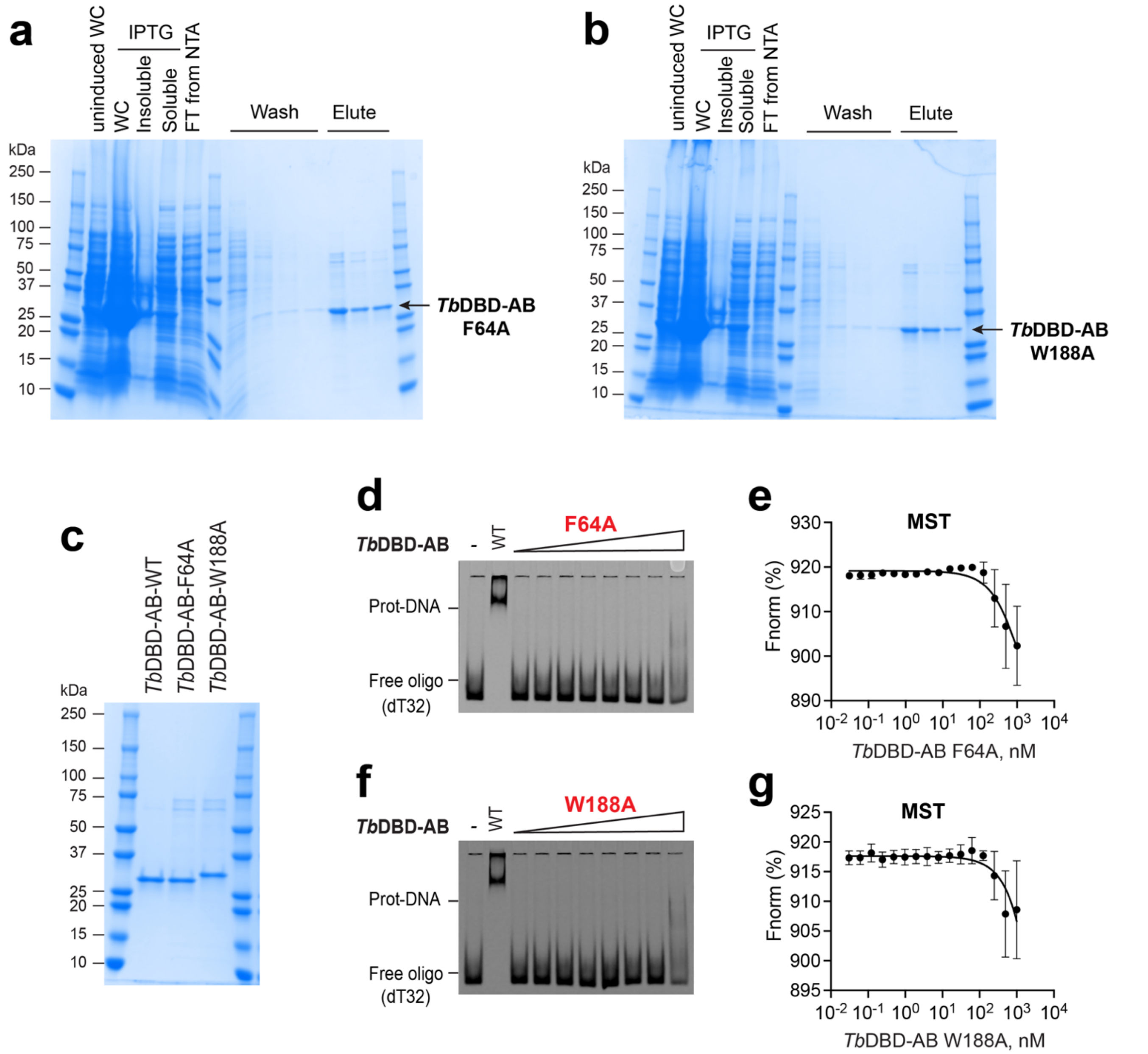

Supplementary Figure 12. ssDNA-binding activity of *Tb*RPA1 DBD-AB F64A and W188A mutants. **a, b** Ni-NTA purification of *Tb*RPA1 DBD-AB F64A (a) and W188A (b) mutant proteins from *E. coli*. Representative SDS-PAGE gels are shown. **c** SDS-PAGE gel showing purified fractions of *Tb*RPA1 DBD-AB WT, F64A, and W188A used in ESMA and MST assays. **d** EMSA with recombinant *Tb*RPA1 DBD-AB F64A. Serial dilutions (0, 1.6, 3.2, 6.25, 12.5, 25, 50 and 100 nM) of *Tb*RPA1 DBD-AB F64A were incubated with 2 nM 5´IRDye800-dT_{32} and samples were analyzed by EMSA. The second lane is wild-type *Tb*RPA1 DBD-AB control (12.5 nM). **e** MST assay with recombinant *Tb*RPA1 DBD-AB F64A. Increasing concentrations of *Tb*RPA1 DBD-AB F64A (2-fold serially diluted starting with a 1000 nM concentration) were incubated with 10 nM 5´Cy5-dT_{32} and RPA-ssDNA binding was examined by MST. **f** EMSA with recombinant *Tb*RPA1 DBD-AB W188A (performed as in d). Three independent EMSA experiments were performed for Supplementary Fig. 12d, f with similar results. **g** MST assay with recombinant *Tb*RPA1 DBD-AB W188A (performed as in e). Three independent MST assays were performed for Supplementary Fig.12e, g (n=3). Error bars indicate mean ±SD. Source data are provided as a Source Data file.

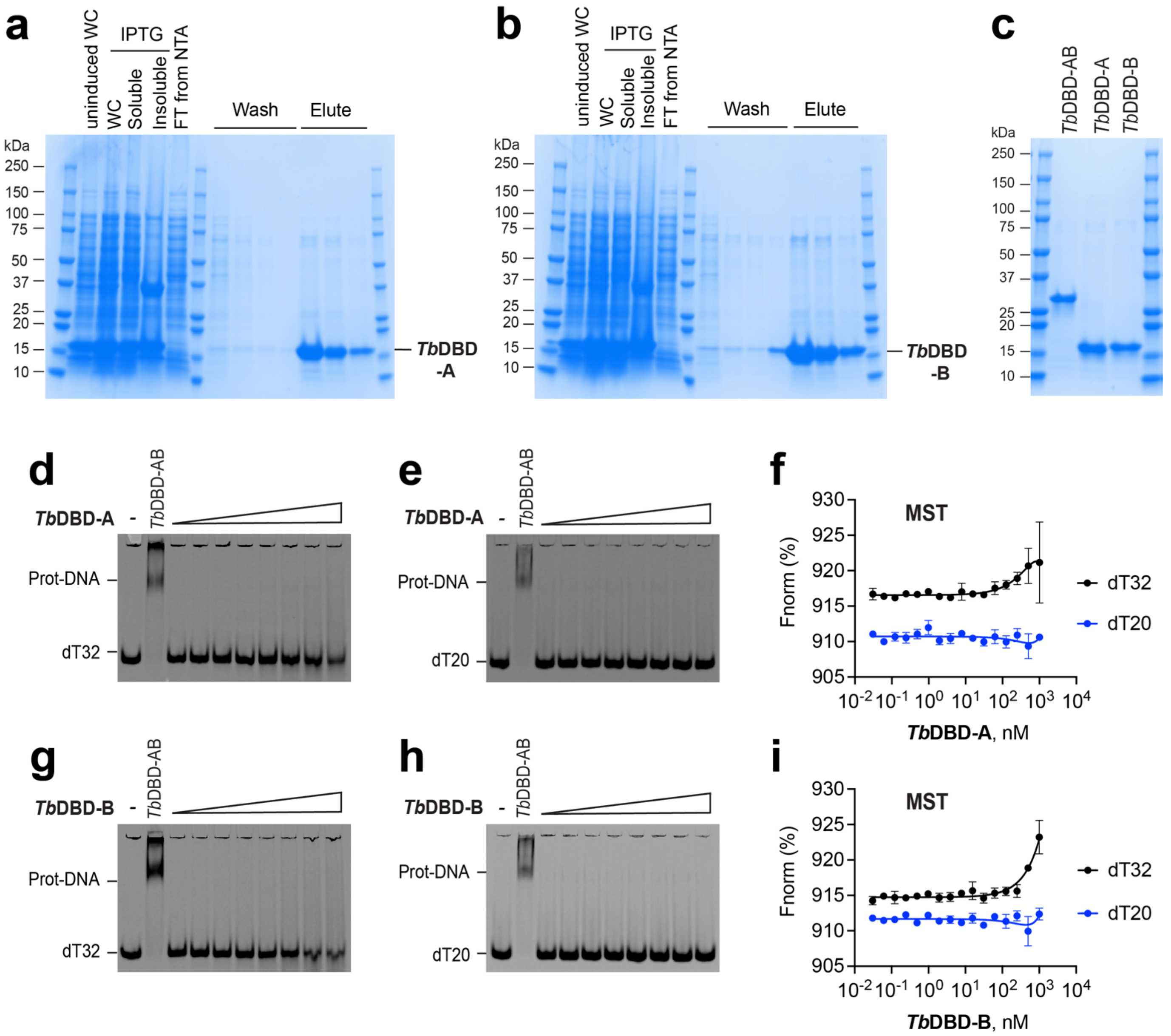

Supplementary Figure 13. ssDNA-binding activity of *Tb*RPA1 DBD-A and *Tb*RPA1 DBD-B. a, b Ni-NTA purification of *Tb*RPA1 DBD-A (a) and DBD-B (b) mutant proteins from *E. coli*. Representative SDS-PAGE gels are shown. **c** SDS-PAGE gel showing purified fractions of *Tb*RPA1 DBD-AB, DBD-A, and DBD-B used in ESMA and MST assays. **d, e** EMSA with recombinant *Tb*RPA1 DBD-A. Serial dilutions (0, 4.67, 9.38, 18.75, 37.5, 75, 150 and 300 nM) of *Tb*RPA1 DBD-A were incubated with 2 nM 5´IRDye800-dT_{32} (d) or dT_{20} (e) and samples were analyzed by EMSA. The second lane is wild-type *Tb*RPA1 DBD-AB control (150 nM). **f** MST assay with recombinant *Tb*RPA1 DBD-A. Increasing concentrations of *Tb*RPA1 DBD-A (2-fold serially diluted starting with a 1000 nM concentration) were incubated with 10 nM 5´Cy5-dT_{32} (black) or dT_{20} (blue) and ssDNA binding was examined by MST. **g, h** EMSA with recombinant *Tb*RPA1 DBD-B (performed as in d and e). **i** MST assay with recombinant *Tb*RPA1 DBD-B (performed as in f). Three independent EMSA experiments were performed for Supplementary Fig. 13d, e, g, h with similar results. Three independent MST assays were performed for Supplementary Fig 13f, i. Error bars indicate mean ±SD. Statistical analysis and plotting of MST data were performed with GraphPad Prism software. K_d values were obtained with a one site binding total curve. Source data are provided as a Source Data file.

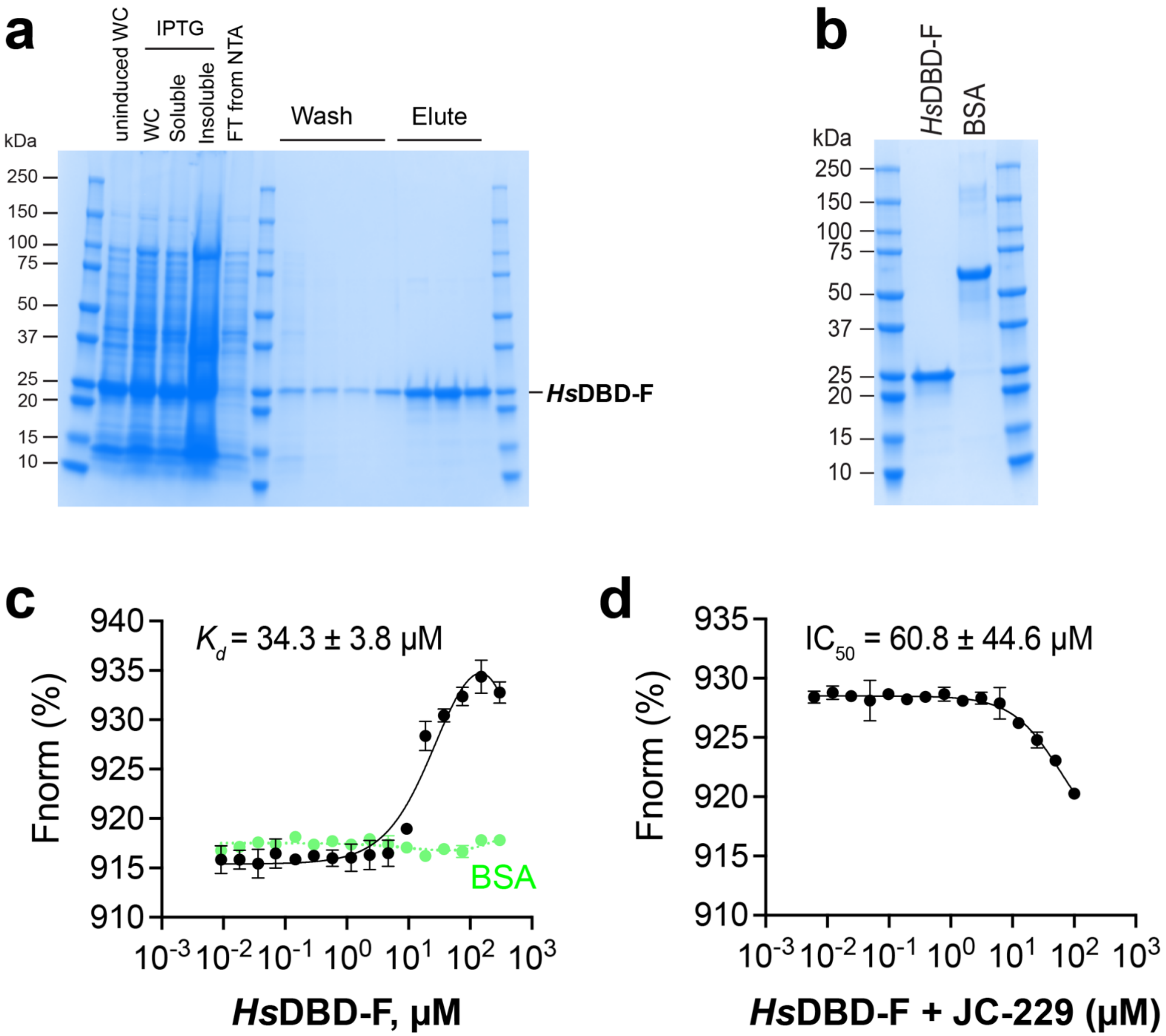

Supplementary Figure 14. *Hs*DBD-F interaction with ATRIP-ε-Acp-Cy5 peptide and JC-229 inhibition. a Ni-NTA purification of *Hs*RPA1 DBD-F protein from *E. coli*. Representative SDS-PAGE gel is shown. **b** An SDS-PAGE gel showing proteins used for MST assays. **c** Binding of recombinant *Hs*DBD-F with ATRIP peptide. *Hs*DBD-F proteins (or BSA) were 2-fold serially diluted starting with a 300 μM concentration and then incubated with 50 nM Cy5-labeled ATRIP peptide. Binding was analyzed by MST. **d** Inhibition of *Hs*DBD-F and ATRIP interaction by JC-229. Serial dilutions of JC-229 were pre-incubated with 20 μM *Hs*DBD-F protein and then with 50 nM Cy5-labeled ATRIP peptide. Three independent MST experiments were performed for Supplementary Fig. 14c, d (n=3). Error bars indicate mean ±SD. Statistical analysis and plotting of MST data were performed with GraphPad Prism software. K_d and IC_{50} values were obtained with a one site binding total and standard 4-PL curve respectively. Source data are provided as a Source Data file.

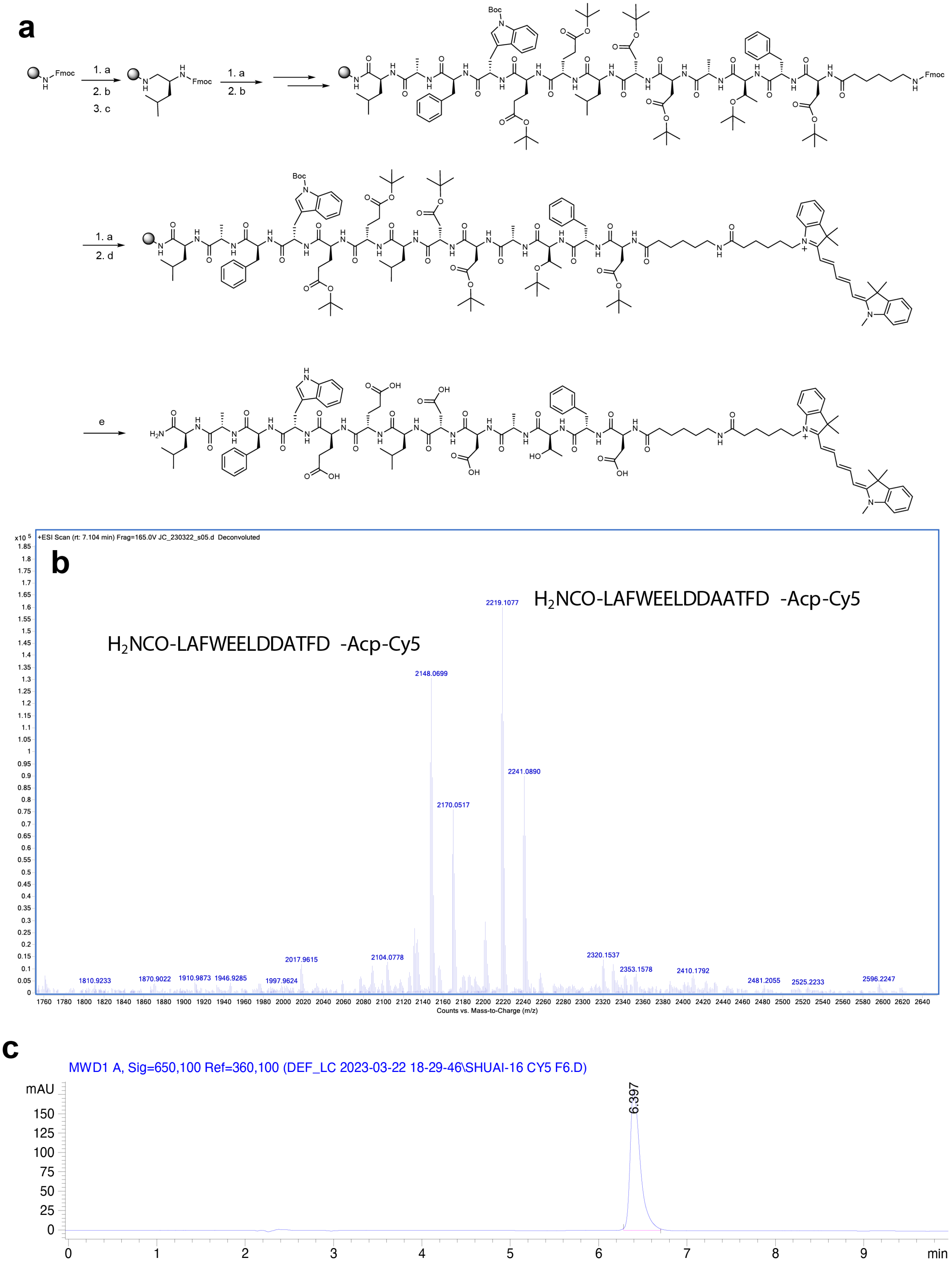
a
1. a
2. b
3. c
1. a
2. b
1. a
2. d
e
Boc
Fmoc
b
+ESI Scan (rt: 7.104 min) Frag=165.0V JC_230322_s05.d Deconvoluted
x10 5
H2NCO-LAFWEELDDAATFD -Acp-Cy5
H2NCO-LAFWEELDDATFD -Acp-Cy5
2219.1077
2148.0699
2241.0890
2170.0517
1810.9233
1870.9022
1910.9873
1946.9285
1997.9624
2017.9615
2104.0778
2320.1537
2353.1578
2410.1792
2481.2055
2525.2233
2596.2247
Counts vs. Mass-to-Charge (m/z)
c
MWD1 A, Sig=650,100 Ref=360,100 (DEF_LC 2023-03-22 18-29-46\SHUAI-16 CY5 F6.D)
mAU
6.397
min

Supplementary Figure 15. Synthesis of ATRIP-ε-Acp-Cy5 peptide (see the methods section for more details). **a** Reagents and conditions: (a) 20% piperidine/DMF(v/v) (25 °C, 2 x 30 min incubation). (b) Fmoc-Leu-OH (5 eq.), HOBt (5 eq.), HATU (5 eq.) in 10% DIPEA/DMF(v/v) (25 °C, 2.5 hours). (c) acetic anhydride/DIPEA/DMF (3:2:5, v/v/v), 25 °C, 30 min; repeat (a) and (b) with the following Fmoc-protected amino acids in sequence: Fmoc-Ala-OH, Fmoc-Phe-OH, Fmoc-Trp(Boc)-OH, Fmoc-Glu(OtBu)-OH, Fmoc-Glu(OtBu)-OH, Fmoc-Leu-OH, Fmoc-Asp(OtBu)-OH, Fmoc-Asp(OtBu)-OH, Fmoc-Ala-OH, Fmoc-Thr(OtBu)-OH, Fmoc-Phe-OH, Fmoc-Asp(OtBu)-OH, and Fmoc-ε-Acp-OH. (d) Cy5-NHS ester (1.2 eq.) in 20% DIPEA/DMF(v/v) (25 °C, 6 hours in dark). (e) TFA/TIPS/H_2O (95:2.5:2.5, v/v/v) (25 °C, 2 hours). **b** High-resolution mass spectrum. There are two major compounds with *m/z* 2,148 and 2,219 (2,170 and 2,241 are the Na adduct to 2,148 and 2,219). 2,148 shows the desired ATRIP peptide, and 2,219 is an ATRIP peptide with one additional Alanine. **c** Liquid Chromatography trace of the synthesized ATRIP peptides. Acp: epsilon-amino-hexanoyl, Cy5: cyanine-5. Source data are provided as a Source Data file.

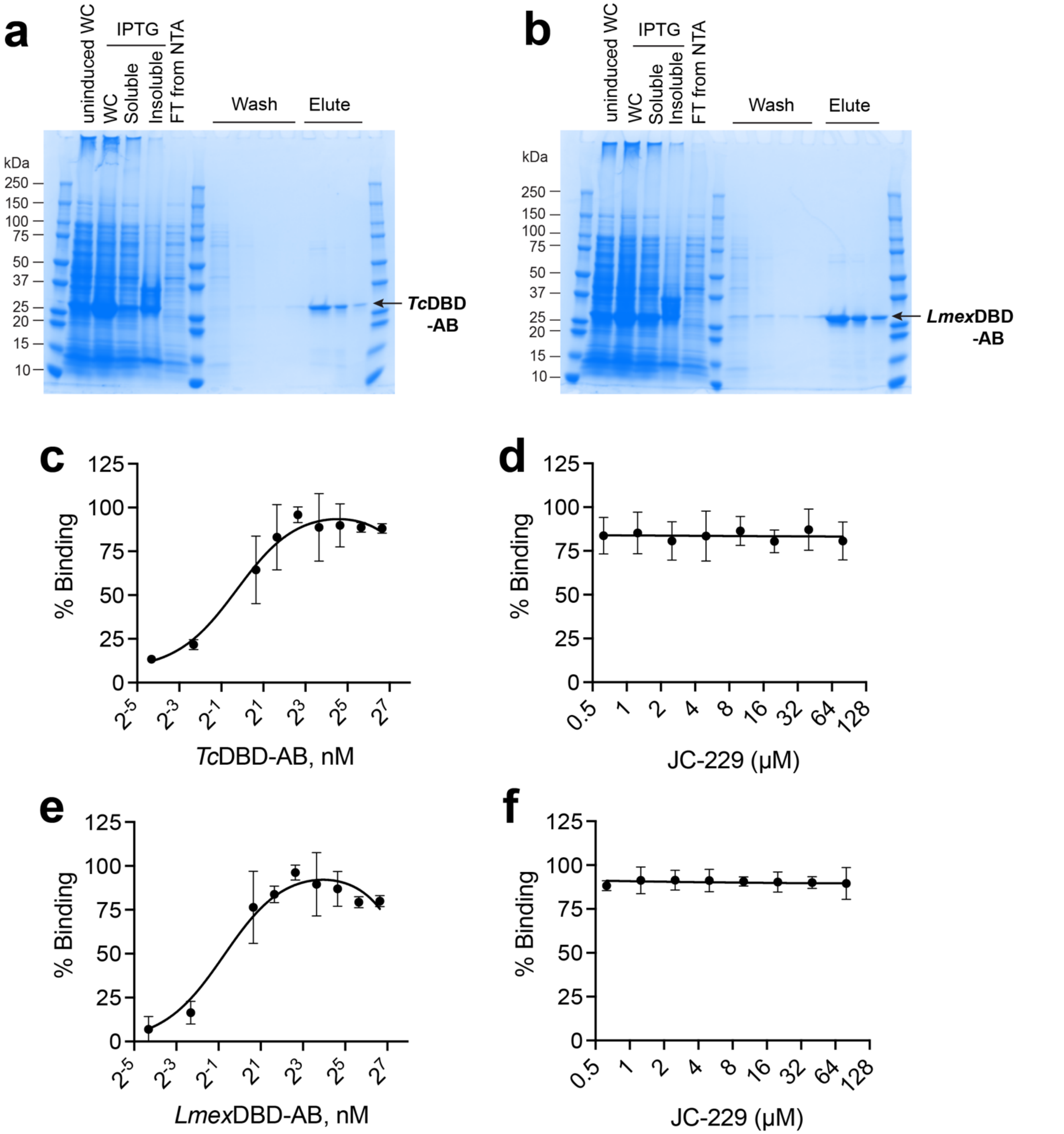

Supplementary Figure 16. Quantification of ssDNA-binding activity of *Tc* and *Lmex*RPA1 DBD-AB, and inhibition by JC-229 by EMSA. a, b Ni-NTA purification of *Tc*RPA1 DBD-AB (a) and *Lmex*RPA1 DBD-AB proteins (b) from *E. coli*. Representative SDS-PAGE gels are shown. **c-f** Quantification of protein-ssDNA complexes in EMSA gels in Fig. 6 (a, b, c, and d, respectively) using ImageJ software. **c** *Tc*RPA1 DBD-AB binding with dT_{32}. **d** Inhibition of *Tc*RPA1 DBD-AB's dT_{32} binding by JC-229. **e** *Lmex*RPA1 DBD-AB binding with dT_{32}. **f** Inhibition of *Lmex*RPA1 DBD-AB's dT_{32} binding by JC-229. EMSA blots from three independent experiments were quantified for Supplementary Fig.16c-f (n=3). Error bars indicate mean ±SD. Source data are provided as a Source Data file.

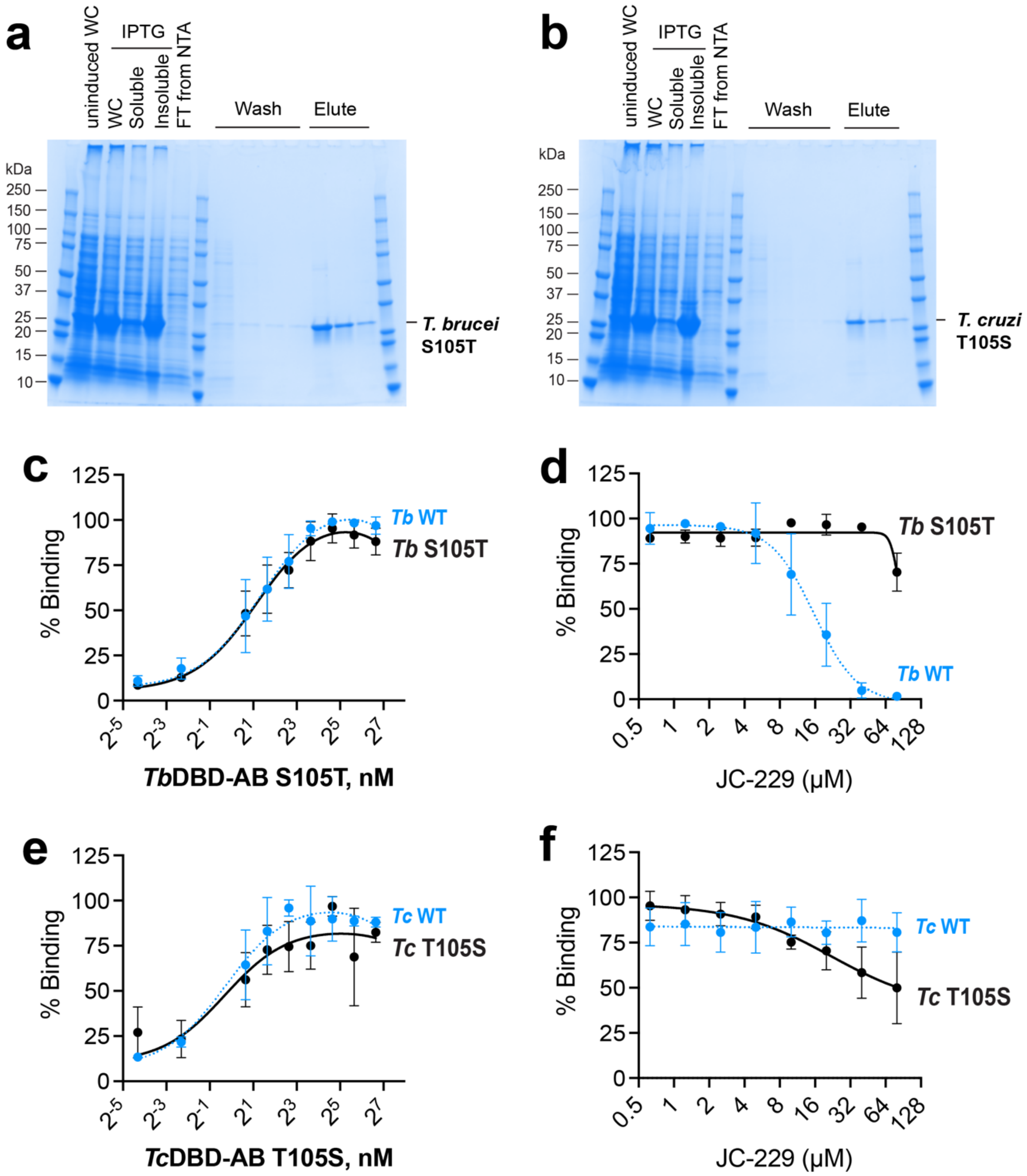

Supplementary Figure 17. Quantification of ssDNA-binding activity of *Tb*RPA1 DBD-AB S105T and *Tc*RPA1 DBD-AB T105S mutants, and inhibition by JC-229 by EMSA. a, b Ni-NTA purification of *Tb*RPA1 DBD-AB S105T (a) and *Tc*RPA1 DBD-AB T105S (b) mutant proteins from *E. coli*. Representative SDS-PAGE gels are shown. **c-f** Quantification of protein-ssDNA complexes in EMSA gels in Fig. 7d, e, h, i using ImageJ software. EMSA blots from three independent experiments were quantified (n=3). Error bars indicate mean ±SD. **c** *Tb*RPA1 DBD-AB S105T mutant's binding with dT_{32} (black). EMSA data of WT *Tb*RPA1 DBD-AB from Supplementary Fig. 9a are overlaid for comparison (light blue). **d** Inhibition of *Tb*RPA1 DBD-AB S105T's dT_{32} binding by JC-229 (black). EMSA data of WT *Tb*RPA1 DBD-AB from Supplementary Fig. 9b are overlaid for comparison (light blue). **e** *Tc*RPA1 DBD-AB T105S mutant's binding with dT_{32}. EMSA data of WT *Tc*RPA1 DBD-AB from Supplementary Fig. 16c are overlaid for comparison (light blue). **f** Inhibition of *Tc*RPA1 DBD-AB T105S mutant's dT_{32} binding by JC-229. EMSA data of WT *Tc*RPA1 DBD-AB from Supplementary Fig. 16d are overlaid for comparison (light blue). Source data are provided as a Source Data file.

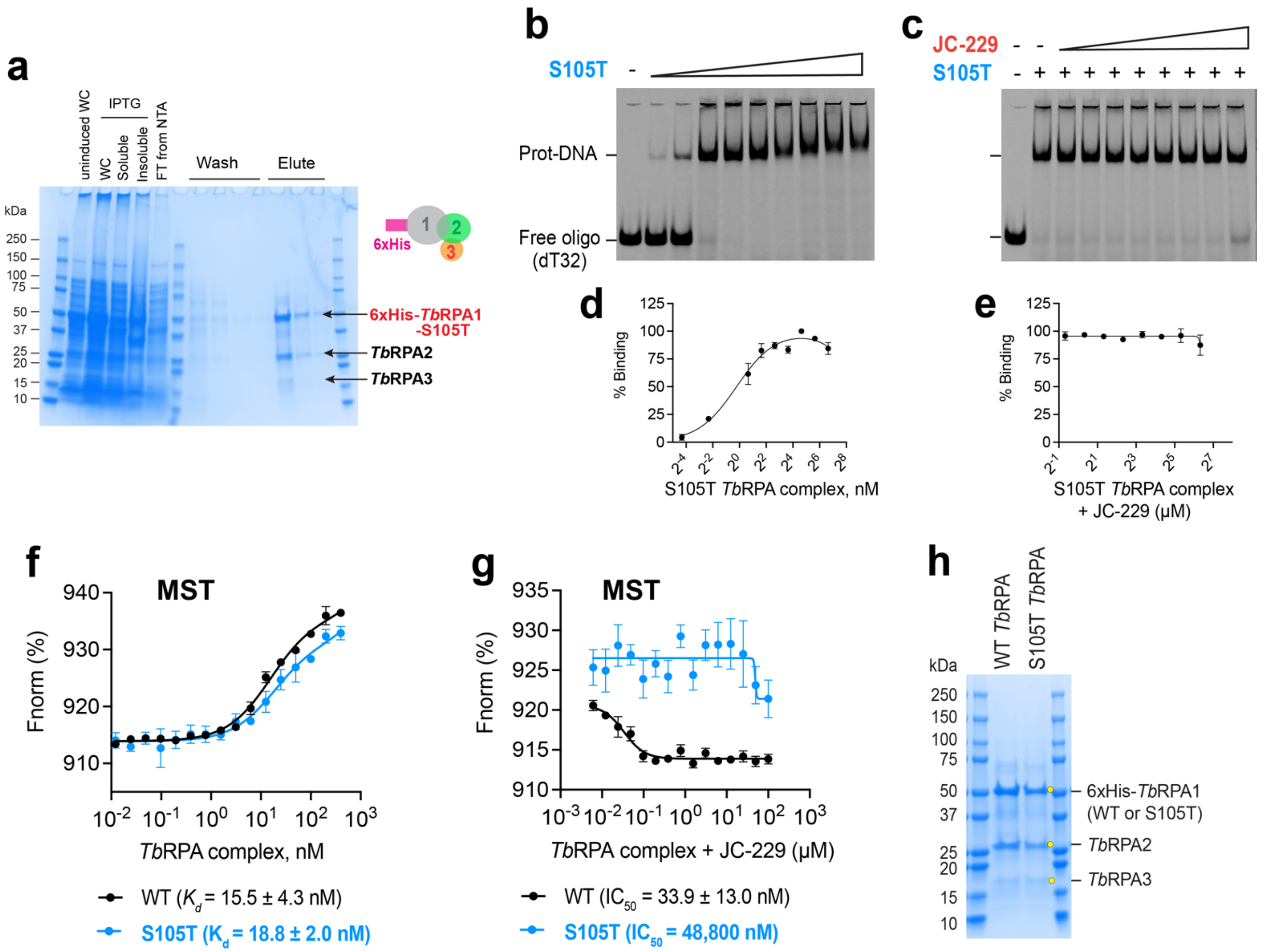

Supplementary Figure 18. ssDNA binding of S105T *Tb*RPA mutant complex and inhibition by JC-229 (EMSA and MST). a Ni-NTA purification of *Tb*RPA S105T mutant complex from *E. coli*. Representative SDS-PAGE gel is shown. **b** EMSA showing ssDNA-binding activity of *Tb*RPA S105T mutant complex. dT_{32} was used as a substrate in all experiments as in Fig. 4. **c** Inhibition of ssDNA-binding activity of *Tb*RPA S105T mutant complex by JC-229. **d, e** Quantification of protein-ssDNA complexes in EMSA gels in Supplementary Fig. 18b, c. Three independent experiments (n=3) were quantified using ImageJ software and plotted. Error bars indicate mean ±SD. **f** MST assay: ssDNA-binding activity of *Tb*RPA S105T mutant complex (blue). *Tb*RPA WT data from Supplementary Fig. 11c (dT_{32}) are overlaid for comparison (black). **g** Inhibition of ssDNA-binding activity of *Tb*RPA S105T mutant by JC-229 (blue). 25 nM of protein was used. *Tb*RPA WT data from Supplementary Fig. 11d (dT_{32}) are overlaid for comparison (black). Three independent MST experiments were performed in Fig.18f, g (n=3). Error bars indicate mean ±SD. Statistical analysis and plotting of MST data were performed with GraphPad Prism software. For *Tb*RPA S105 mutant, K_d was obtained with one site binding total and IC_{50} was obtained with [Inhibitor] vs. response, variable slope (four parameters). **h** SDS-PAGE gel showing purified fractions used for EMSA and MST assays. Source data are provided as a Source Data file.

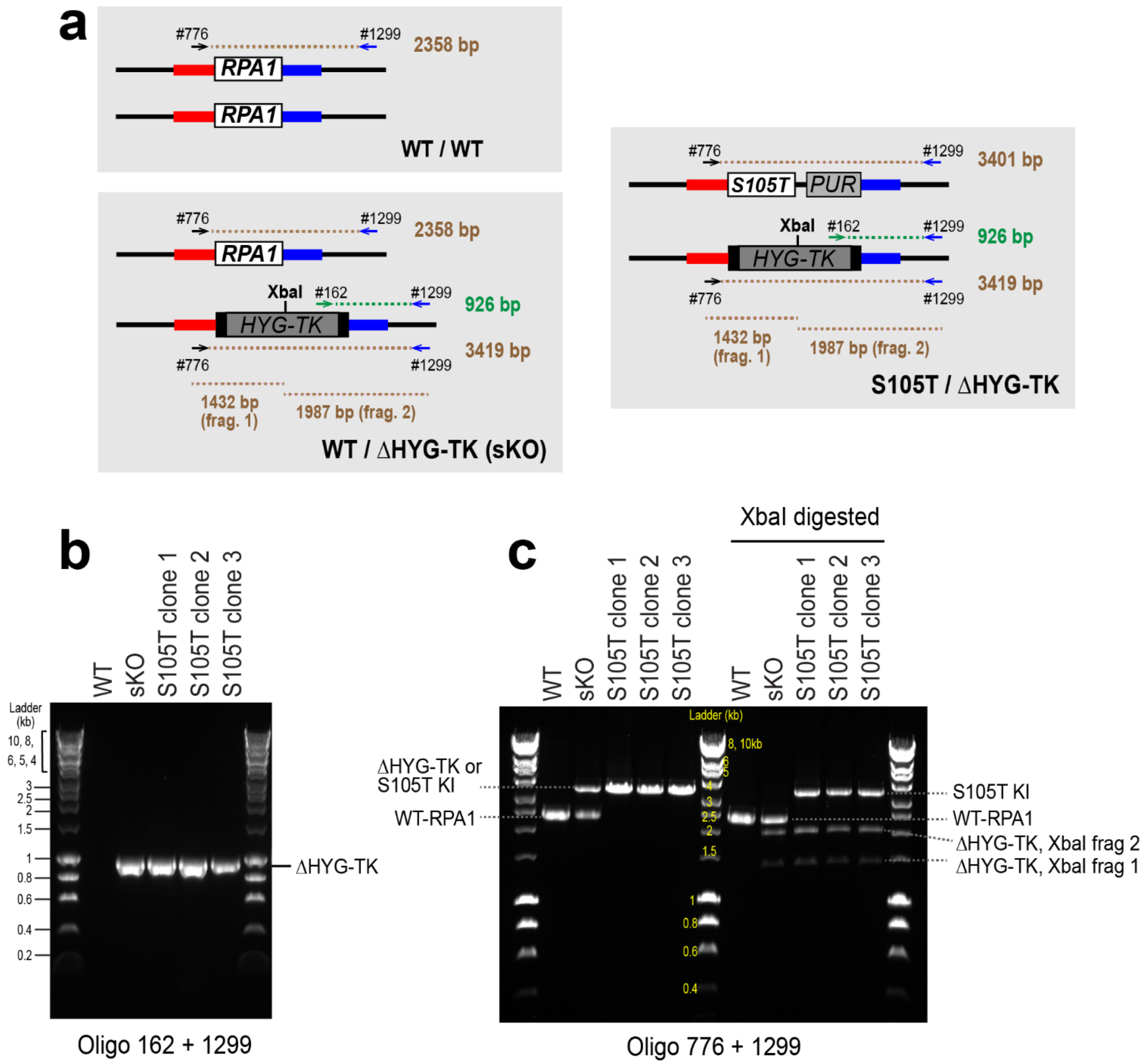

Supplementary Figure 19. PCR genotyping of *T. brucei* mutant clones containing *Tb*RPA1-S105T mutation. a PCR genotyping strategy for *Tb*RPA1 alleles in the strains of WT (WT/WT), single knock-out (sKO, WT/ΔHYG-TK) and S105T mutant (S105T/ΔHYG-TK). The sizes of the PCR products amplified with oligos 776 and 1299 will be similar between *Tb*RPA1ΔHYG-TK and *Tb*RPA1-S105T alleles (3,419 bp and 3,401 bp respectively). The amplicon from *Tb*RPA1ΔHYG-TK allele should have an XbaI restriction enzyme site within the HYG-TK gene, while no XbaI is present in the amplicon from S105T allele. Thus, two amplicons can be distinguished by XbaI digestion. **b** PCR genotyping confirming the presence of *Tb*RPA1 KO allele in the sKO and three of the S105T mutant clones. **c** PCR genotyping confirming the genotypes of sKO and S105T mutant clones. *Tb*RPA1 WT allele was amplified in the WT strain (2,358 bp). To distinguish the KO and S105T alleles, XbaI digestion was performed. As expected, S105T amplicon (3,401 bp) was undigested and KO amplicon (3,419bp) yielded products of 1,987 bp and 1,432 bp after XbaI enzyme digestion. Source data are provided as a Source Data file.

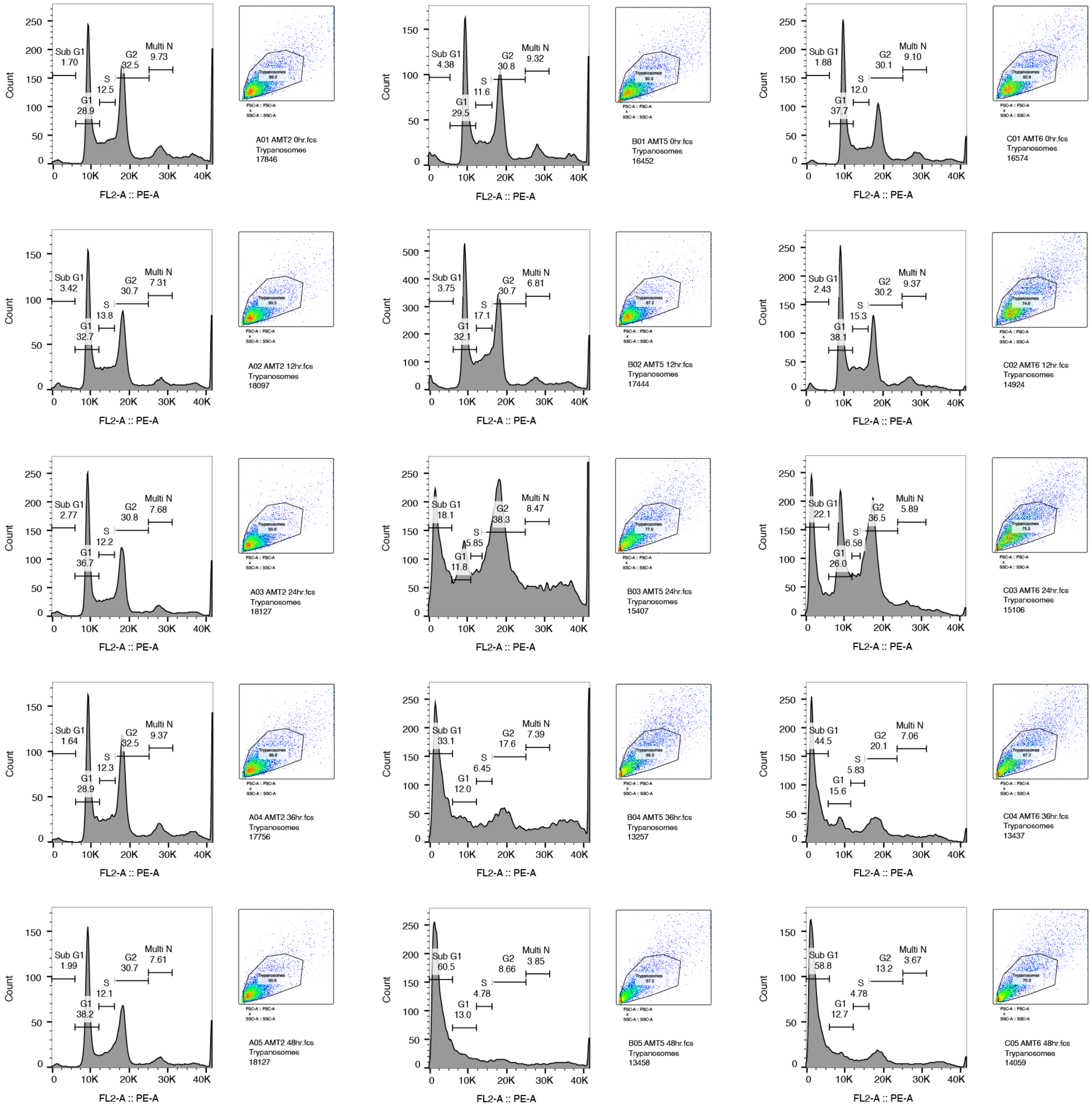

Supplementary Figure 20. Gating strategies used to analyze the cell-cycle profiles of *Tb*RPA1-depleted trypanosome cells (Fig. 2d). Gatings in the cell-cycle histogram show the percentage of cells in Sub-G1, G1, S, G2 or multinucleated (Multi N). Scatter plots show trypanosome cell gating in each sample.

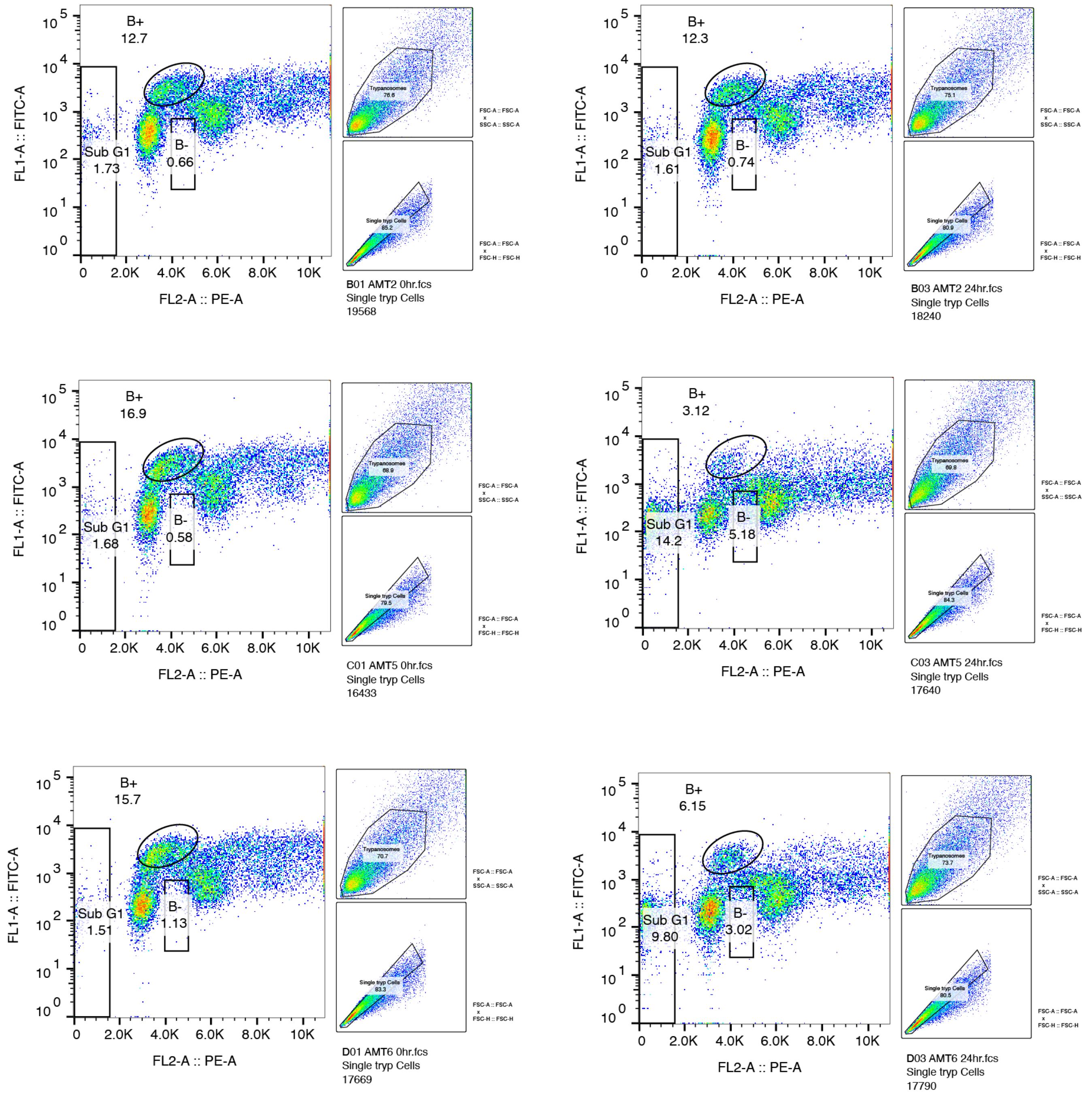

Supplementary Figure 21. Gating strategies used to analyze BrdU-pulse labeled WT and *Tb*RPA1-depleted trypanosome cells (Fig. 2e). Sub-G1, BrdU-positive ($BrdU^+$) and BrdU-negative ($BrdU^-$) cells are gated in the scatter plots on the left. Two plots on the right show trypanosome cell and single cell gating strategies.

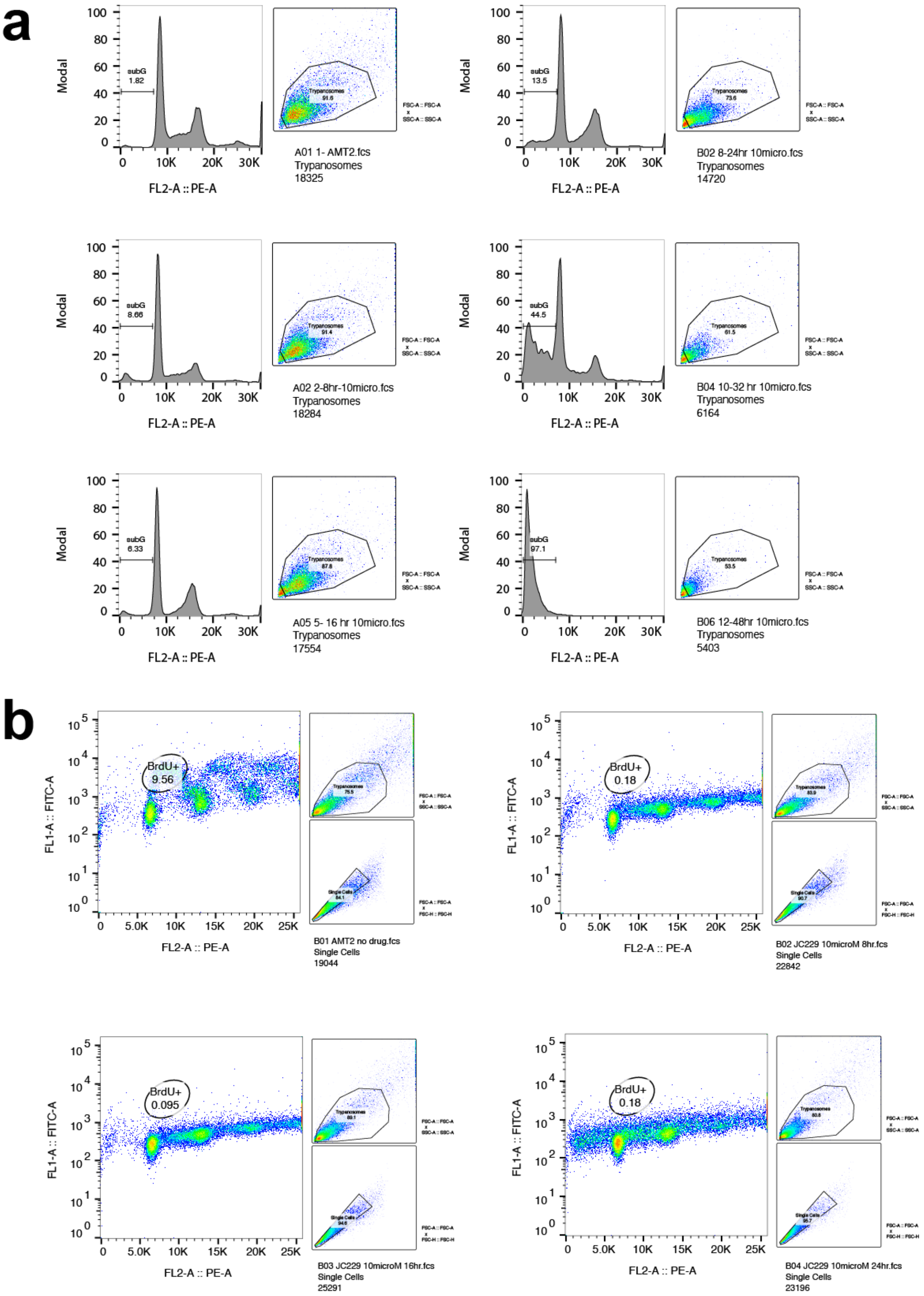

Supplementary Figure 22. Gating strategies used to analyze the cell-cycle profiles of JC-229-treated trypanosome cells (Fig. 3a) and BrdU-pulse labeling of JC-229-treated trypanosome cells (Fig. 3b). **a** Gatings in the cell-cycle histogram show the percentage of cells in Sub-G1. Scatter plots show trypanosome cell gating in each sample. **b** BrdU-pulse labeling. BrdU-positive ($BrdU^+$) cells are gated in the scatter plots on the left. Two plots on the right show trypanosome cell and single cell gating strategies.

Supplementary References

1. Alsford, S. et al. High-throughput phenotyping using parallel sequencing of RNA interference targets in the African trypanosome. *Genome Res* **21**, 915-924 (2011).
2. Wirtz, E., Leal, S., Ochatt, C. & Cross, G. A. M. A tightly regulated inducible expression system for dominant negative approaches in *Trypanosoma brucei*. *Molecular & Biochemical Parasitology* **99**, 89-101 (1999).
3. Schulz, D., Zaringhalam, M., Papavasiliou, F. N. & Kim, H. S. Base J and H3.V Regulate Transcriptional Termination in Trypanosoma brucei. *PLoS Genet* **12**, e1005762 (2016).
4. Henricksen, L. A., Umbricht, C. B. & Wold, M. S. Recombinant replication protein A: expression, complex formation, and functional characterization. *J Biol Chem* **269**, 11121-11132 (1994).
