# Supplementary material for: Identification of a small-molecule inhibitor that selectively blocks DNA-binding by Trypanosoma brucei replication protein A1
Source: Nat Commun. 2023 Jul 20;14:4390. doi: 10.1038/s41467-023-39839-x (PMC10359466; doi:10.1038/s41467-023-39839-x)
Supplement: Supplementary file 3 — Reporting summary [file 41467_2023_39839_MOESM3_ESM.pdf]

nature portfolio

Corresponding author(s): Hee-Sook Kim, Jun yong Choi

Last updated by author(s): Jun 12, 2023

Reporting Summary

Nature Portfolio wishes to improve the reproducibility of the work that we publish. This form provides structure for consistency and transparency in reporting. For further information on Nature Portfolio policies, see our Editorial Policies and the Editorial Policy Checklist.

Statistics

For all statistical analyses, confirm that the following items are present in the figure legend, table legend, main text, or Methods section.

n/a	Confirmed	
☐	☒	The exact sample size (n) for each experimental group/condition, given as a discrete number and unit of measurement
☐	☒	A statement on whether measurements were taken from distinct samples or whether the same sample was measured repeatedly
☐	☒	The statistical test(s) used AND whether they are one- or two-sided<br>*Only common tests should be described solely by name; describe more complex techniques in the Methods section.*
☒	☐	A description of all covariates tested
☒	☐	A description of any assumptions or corrections, such as tests of normality and adjustment for multiple comparisons
☐	☒	A full description of the statistical parameters including central tendency (e.g. means) or other basic estimates (e.g. regression coefficient) AND variation (e.g. standard deviation) or associated estimates of uncertainty (e.g. confidence intervals)
☐	☒	For null hypothesis testing, the test statistic (e.g. F, t, r) with confidence intervals, effect sizes, degrees of freedom and P value noted<br>*Give P values as exact values whenever suitable.*
☒	☐	For Bayesian analysis, information on the choice of priors and Markov chain Monte Carlo settings
☒	☐	For hierarchical and complex designs, identification of the appropriate level for tests and full reporting of outcomes
☒	☐	Estimates of effect sizes (e.g. Cohen's d, Pearson's r), indicating how they were calculated

Our web collection on statistics for biologists contains articles on many of the points above.

Software and code

Policy information about availability of computer code

Data collection	Flow cytometry data were collected from BD FACS Via and BD FACSDiva software (BD Biosciences).<br>MicroScale Thermophoresis data were obtained using Monolith NT.115 instrument.<br>EMSA data were obtained using LI-CORimaging system.<br>Fluorescence readings for AlamarBlue assay were obtained using Synergy Hl microplate reader (BioTek).<br>Cell images (DIC and DAPI stained) were obtained from EVOS M5000 microscope.<br>The homology model of TbRPAl was obtained from the protein structure homology-modeling server (SWISS-MODEL), and the structure was built based on the 4gop.l.C structure.<br>Induced Fit Docking module in Schrodinger Small Molecule Drug Discovery Suite 2020-2 was used to generate the model structure of the TbRPAl - JC-229 complex.<br>lH and 13C NMR spectra were recorded on a Bruker 400 MHz and 101 MHz instrument, respectively. Mass Spectra were collected over m/z 200-1200 and recorded on Advion Mass Express CMS v6.2.22.l.<br>Mass spectrometry was performed using a Q Exactive HF tandem mass spectrometer coupled to a Dionex Ultimate 3000 RLSCnano system (Thermo Scientific). The LC-MS/MS peak list was generated by Thermo Proteome Discoverer (v.2.1) into Mascot Generic Format (MGS).<br>Mass photometry data was acquired on a Refeyn OneMP instrument (Refeyn Ltd).<br>ATRIP-ε-Acp-Cy5 probe was characterized by high-resolution Mass Spectrometer (HRMS) and analytical Liquid Chromatography (LC) at λ = 650 nm: HRMS (ESI-TOF) m/z calculated for C111H147N18O26+ [M]+ 2148.0728, found 2148.0699.
Data analysis	Flow cytometry data were analyzed using FlowJo software (version 10).<br>Images were analyzed using ImageJ (version 1.53) and Adobe Photoshop (version 24.5.0).<br>Data plotting and statistical analyses were performed using GraphPad Prism (version 9.3.1).

The model structures were visually analyzed in Maestro v12.4, and structural images were generated using PyMOL 2.0.7.
MestReNova v14.1.0 software was used to analyze NMR spectroscopic data.
Advion Data Express v6.2.22.1 was used to analyze Mass spectroscopic data.
Mass photometry data were analyzed using Refeyn AcquireMP (v. 2022 R1; Refeyn Ltd) and Refeyn DiscoverMP (v. 2022 R1; Refeyn Ltd).

For manuscripts utilizing custom algorithms or software that are central to the research but not yet described in published literature, software must be made available to editors and reviewers. We strongly encourage code deposition in a community repository (e.g. GitHub). See the Nature Portfolio guidelines for submitting code & software for further information.

Data

Policy information about availability of data

All manuscripts must include a data availability statement. This statement should provide the following information, where applicable:

- Accession codes, unique identifiers, or web links for publicly available datasets
- A description of any restrictions on data availability
- For clinical datasets or third party data, please ensure that the statement adheres to our policy

All the data are available within the manuscript, Supplementary Information and Source File.
LC-MS/MS data is accessible in the PreoteomeXchange Consortium-PRIDE repository database with accession code PXD042808.

Human research participants

Policy information about studies involving human research participants and Sex and Gender in Research.

Reporting on sex and gender	N/A
Population characteristics	N/A
Recruitment	N/A
Ethics oversight	N/A

Note that full information on the approval of the study protocol must also be provided in the manuscript.

Field-specific reporting

Please select the one below that is the best fit for your research. If you are not sure, read the appropriate sections before making your selection.

☒ Life sciences ☐ Behavioural & social sciences ☐ Ecological, evolutionary & environmental sciences

For a reference copy of the document with all sections, see nature.com/documents/nr-reporting-summary-flat.pdf

Life sciences study design

All studies must disclose on these points even when the disclosure is negative.

Sample size	Sample size was determined by data availability.
Data exclusions	No data were excluded.
Replication	All results were reproducible, performed independently at least 3 times and mean value was used.
Randomization	All data were analyzed in an unbiased way.
Blinding	Blinding was not used because no bias or randomization was involved in the study.

Reporting for specific materials, systems and methods

We require information from authors about some types of materials, experimental systems and methods used in many studies. Here, indicate whether each material, system or method listed is relevant to your study. If you are not sure if a list item applies to your research, read the appropriate section before selecting a response.

Materials & experimental systems

n/a	Involved in the study
☐	☒ Antibodies
☐	☒ Eukaryotic cell lines
☒	☐ Palaeontology and archaeology
☒	☐ Animals and other organisms
☒	☐ Clinical data
☒	☐ Dual use research of concern

Methods

n/a	Involved in the study
☒	☐ ChIP-seq
☐	☒ Flow cytometry
☒	☐ MRI-based neuroimaging

Antibodies

Antibodies used	We have used our own rabbit polyclonal gamma H2A antibody [generated by ABclonal, ID E16070 (P)] ; Rabbit polyclonal RPAl antibody (ID:21371) was a gift from Dr. Li (Cleavland State University) as mentioned in the acknowledgments; Mouse monoclonal VSG3 [Catalog/Clone VSG224-11D6, Antibody and Bioresource Core facility at MSKCC], mouse Tubulin [Sigma, Catalog T7451, Clone 6-11B-1, LOT number 0000149704] and mouse BrdU [BD Pharmingen, Catalog 555627, lot 6084615] antibodies were obtained commercially. Donkey anti-mouse A488 [Invitrogen, catalog A-21202, lot 94-C2-1], rabbit HRP [GE healthcare, catalog NA934V, lot 17640116], mouse HRP [GE healthcare, catalog NA931V, lot lot 17041904] secondary antibodies were used.
Validation	Mouse VSG3 antibody in PMID 29018220 & PMID 34722526; Mouse Tubulin antibody in PMID 34722526. BrdU antibody: PMID 34722526

Eukaryotic cell lines

Policy information about cell lines and Sex and Gender in Research

Cell line source(s)	Trypanosoma brucei brucei Lister 427 strain (Single Marker, SM background) originally from George Cross Laboratory, Rockefeller University, in Methods, page 17. HSTB-904 was generated in SM background, Methods, page 17.<br>Trypanosoma brucei brucei Lister 427 strain (2T1) from David Horn, University of Dundee, in Methods, page 17 (originally from George Cross Laboratory, Rockefeller University).<br>HeLa (CRM-CCL-2) and HEK293 (CRL-3216) cell lines are commercially available from ATCC and were kindly gifted by Suzuki lab, Rutgers University.
Authentication	RNA-seq
Mycoplasma contamination	We did not test Mycoplasma contamination.
Commonly misidentified lines (See ICLAC register)	No commonly misidentified cell lines were used.

Flow Cytometry

Plots

Confirm that:

☒ The axis labels state the marker and fluorochrome used (e.g. CD4-FITC).

☒ The axis scales are clearly visible. Include numbers along axes only for bottom left plot of group (a 'group' is an analysis of identical markers).

☒ All plots are contour plots with outliers or pseudocolor plots.

☒ A numerical value for number of cells or percentage (with statistics) is provided.

Methodology

Sample preparation	See section "Cell-cycle analysis and BrdU pulse experiments using flow cytometry" in Methods, page 19.
Instrument	BD-FACSVia.
Software	FACSDiva and FlowJo software (V.10) was used for analysis.
Cell population abundance	N/A. We used a single cell type (T. brucei).

Gating strategy

For cell cycle analysis, trypanosome cells were gated via forward and side scatter (FSC vs SSC) to eliminate cell debris. For BrdU pulse, trypanosome cells were gated using FSC vs SSC and then gated via FSC-A vs FSC-H for single cell gating.

☒ Tick this box to confirm that a figure exemplifying the gating strategy is provided in the Supplementary Information.
